# Enabling quaternion derivatives: the generalized HR calculus

**DOI:** 10.1098/rsos.150255

**Published:** 2015-08-26

**Authors:** Dongpo Xu, Cyrus Jahanchahi, Clive C. Took, Danilo P. Mandic

**Affiliations:** 1 School of Mathematics and Statistics, Northeast Normal University, Changchun 130024, People's Republic of China; 2 Department of Electrical and Electronic Engineering, Imperial College London, London SW7 2AZ, UK; 3 Department of Computing, University of Surrey, Guildford GU2 7XH, UK

**Keywords:** generalized HR calculus, non-analytic quaternion function, nonlinear quaternion functions, quaternion derivatives, quaternion least mean square

## Abstract

Quaternion derivatives exist only for a very restricted class of analytic (regular) functions; however, in many applications, functions of interest are real-valued and hence not analytic, a typical case being the standard real mean square error objective function. The recent HR calculus is a step forward and provides a way to calculate derivatives and gradients of both analytic and non-analytic functions of quaternion variables; however, the HR calculus can become cumbersome in complex optimization problems due to the lack of rigorous product and chain rules, a consequence of the non-commutativity of quaternion algebra. To address this issue, we introduce the generalized HR (GHR) derivatives which employ quaternion rotations in a general orthogonal system and provide the left- and right-hand versions of the quaternion derivative of general functions. The GHR calculus also solves the long-standing problems of product and chain rules, mean-value theorem and Taylor's theorem in the quaternion field. At the core of the proposed GHR calculus is quaternion rotation, which makes it possible to extend the principle to other functional calculi in non-commutative settings. Examples in statistical learning theory and adaptive signal processing support the analysis.

## 1. Introduction

Quaternions have become a standard tool in many modern areas, including image processing [1,2], aerospace and satellite tracking [3], modelling of wind profile in renewable energy [4] and in the processing of polarized waves [5,6]. Compared to real vector algebra, quaternion algebra [7] has been shown to both reduce the number of parameters in the modelling and offer advantages in terms of functional simplicity and accuracy [8,9]. The most common optimization approach in applications is based on the gradient of the objective function; one such algorithm is the quaternion least mean square (QLMS) [4]. The objective functions in practical applications are typically based on the mean square error (MSE), a real function of quaternion variables, and are thus not analytic according to standard quaternion analysis [10–12]. This is a major obstacle to a more widespread use of quaternions in learning systems.

The existing ways to find the derivative of a real function *f*(*q*) with respect to the unknown quaternion variable *q* are:

— *The pseudo-derivative*, which considers *f* as a function of the four real components *q*_*a*_,*q*_*b*_,*q*_*c*_ and *q*_*d*_ of the quaternion variable *q*, and then takes componentwise real derivatives with respect to the real variables *q*_*a*_,*q*_*b*_,*q*_*c*_ and *q*_*d*_. In other words, the real-valued function f:H→R is treated as a real differentiable mapping between $R^{4}$ and R. This leads to unnecessarily long expressions and is especially cumbersome and tedious in complex optimization problems and when dealing with nonlinear functions.
— *The HR calculus*, which is compact and elegant [13], as it finds formal derivatives of *f* with respect to the quaternion variables and their involutions in a direct way. This applies to both real functions of quaternion variables and nonlinear functions. This approach is based on the differentials of *q*,*q*^*i*^,*q*^*j*^,*q*^*k*^, which are independent in the quaternion field, as shown in lemmas A.1 and A.2. The advantage of using HR derivatives is that the computations and analysis are kept in the quaternion domain rather than using quaternion-to-real transformations, and many algorithms can be readily extended from the complex to the quaternion domain.

Although the HR calculus is a significant step forward, the product and chain rules are not defined within the HR calculus, which complicates the calculation of derivatives of, for example, nonlinear quaternion functions. Other functional calculi [10–12,14] in quaternion analysis similarly suffer from this obstacle.

The aim of this work is to revisit the HR calculus [13] and to equip it with the product rule and chain rule in order to solve these long-standing problems in quaternion calculus. Motivated by the complex CR calculus [15–17], we first consider a general orthogonal system which, in conjunction with the HR calculus, introduces the generalized HR (GHR) calculus. The GHR calculus comprises both the left- and right-hand versions of quaternion derivative; these are necessary to consider due to non-commutativity of quaternion product. In particular, we show that for real functions of quaternion variables, such as the standard MSE objective function, the left and right GHR derivatives are identical. An important consequence of this property is that within the GHR calculus, the choice of the left/right GHR derivative is irrelevant for practical applications of quaternion optimization; this is currently a major source of confusion in the quaternion community. Another consequence of the novel product rule is that it not only enables the calculation of the GHR derivatives for general functions of quaternion variables, but also it is generic—if one function within the product is real-valued, this novel product rule degenerates into the traditional product rule, as shown in corollary 4.11. A family of chain rules is also introduced in order to calculate the derivatives of nonlinear functions of quaternion variables, which include complex- and real-valued functions as degenerate quaternion functions. Since at the core of the GHR calculus is the quaternion rotation, this approach can be naturally extended to other functional calculi in non-commutative settings. Finally, we revisit two fundamental theorems in quaternion calculus—the quaternion mean value theorem and quaternion Taylor's theorem—and derive them in a compact and generic form, based on the GHR derivatives. The GHR calculus therefore provides a solution to some long-standing mathematical problems [18] and promises a tool for a more widespread use of quaternions in practical applications. Illustrative examples in statistical signal processing support the analysis.

## 2. Background on quaternions

### 2.1 Quaternion algebra

Quaternions are an associative but not commutative algebra over R, defined as

2.1 $$H=\mathrm{span}\{1,i,j,k\}≜\{q_{a}+iq_{b}+jq_{c}+kq_{d}\;|\;q_{a},q_{b},q_{c},q_{d}\in R\},$$

where {1,*i*,*j*,*k*} is a basis of H, and the imaginary units *i*,*j* and *k* satisfy *i*^2^=*j*^2^=*k*^2^=*ijk*=−1, which implies *ij*=*k*=−*ji*, *jk*=*i*=−*kj*, *ki*=*j*=−*ik*. For any quaternion *q*=*q*_*a*_+*iq*_*b*_+*jq*_*c*_+*kq*_*d*_=*S*_*q*_+*V*_*q*_, the scalar (real) part is denoted by $q_{a}=S_{q}=R(q)$, whereas the vector part $V_{q}=I(q)=iq_{b}+jq_{c}+kq_{d}$ spans the three imaginary parts. For p,q∈H, the quaternion product is given by *pq*=*S*_*p*_*S*_*q*_−*V*_*p*_⋅*V*_*q*_+*S*_*p*_*V*_*q*_+*S*_*q*_*V*_*p*_+*V*_*p*_×*V*_*q*_, where the symbols ′⋅′ and ′×′ denote, respectively, the standard inner product and vector product. The presence of vector product makes the quaternion product non-commutative, i.e. in general for p,q∈H, *pq*≠*qp*. The conjugate of a quaternion *q* is defined as q*=*S*_*q*_−*V*_*q*_, while the conjugate of a quaternion product satisfies (*pq*)*=q*p*. The modulus of a quaternion is defined as $|q|=\sqrt{qq^{*}}$, and thus |*pq*|=|*p*||*q*|. The inverse of a quaternion *q*≠0 is *q*^−1^=q*/|*q*|^2^ which yields an important consequence (*pq*)^−1^=*q*^−1^*p*^−1^. If |*q*|=1, we call *q* a *unit* quaternion. A quaternion *q* is said to be *pure* if R(q)=0. For pure quaternions, q*=−*q* and *q*^2^=−|*q*|^2^. Thus, a *pure unit* quaternion is a square root of −1; examples are the imaginary units *i*,*j* and *k*.

Quaternions can also be written in the *polar* form $q=|q|(\cos\theta +\hat{q}\sin\theta )$, where $\hat{q}=Vq/|Vq|$ is a pure unit quaternion and θ=arccos⁡(R(q)/|q|)∈R is the angle (or argument) of the quaternion. We shall next introduce the quaternion rotation and involution operators.

**Definition 2.1 (quaternion rotation [19])**

For any quaternion *q*, the transformation

2.2 $$q^{\mu }≜\mu q\mu^{-1}$$

geometrically describes a three-dimensional rotation of the vector part of *q* by an angle 2*θ* about the vector part of *μ*, where $\mu =|\mu |(\cos\theta +\hat{\mu }\sin\theta )$ is any non-zero quaternion.

Properties of the quaternion rotation (see [6,20] used in this work) are

2.3 $${\left(pq\right)}^{\mu }=p^{\mu }q^{\mu },\;pq=q^{p}p=qp^{\left(q^{*}\right)},\;\forall \;p,q\in H$$

and

2.4 $$q^{\mu \nu }={\left(q^{\nu }\right)}^{\mu },\;q^{\mu *}≜{\left(q^{*}\right)}^{\mu }={\left(q^{\mu }\right)}^{*}≜q^{*\mu },\;\forall \;\nu ,\mu \in H.$$

Note that the representation in (2.1) can be generalized to a general orthogonal basis {1,*i*^*μ*^,*j*^*μ*^,*k*^*μ*^}, where the following properties hold [19]:

2.5 $$i^{\mu }i^{\mu }=j^{\mu }j^{\mu }=k^{\mu }k^{\mu }=i^{\mu }j^{\mu }k^{\mu }=-1.$$

**Definition 2.2 (quaternion involution [21])**

The involution of a quaternion *q* around a pure unit quaternion *η* is given by

$$q^{\eta }=\eta q\eta^{-1}=\eta q\eta^{*}=-\eta q\eta$$

and represents a rotation of *q* about *η* by *π*.

Of particular interest to this work are quaternion involutions around the imaginary units *i*,*j*,*k*, given by [21]

2.6 $$\begin{array}{rl} q & =q_{a}+iq_{b}+jq_{c}+kq_{d},\;q^{i}=-iqi=q_{a}+iq_{b}-jq_{c}-kq_{d}\\ q^{j} & =-jqj=q_{a}-iq_{b}+jq_{c}-kq_{d},\;q^{k}=-kqk=q_{a}-iq_{b}-jq_{c}+kq_{d}, \end{array}\}$$

which allows us to express the four real-valued components of a quaternion *q* as [13,21]

2.7 $$\begin{array}{rl} q_{a} & =\frac{1}{4}(q+q^{i}+q^{j}+q^{k}),\;q_{b}=\frac{1}{4i}(q+q^{i}-q^{j}-q^{k}), \end{array}$$

2.8 $$\begin{array}{rl} q_{c} & =\frac{1}{4j}(q-q^{i}+q^{j}-q^{k}),\;q_{d}=\frac{1}{4k}(q-q^{i}-q^{j}+q^{k}). \end{array}$$

This is analogous to the complex case, where $x=\frac{1}{2}(z+z^{*})$ and *y*=−(*i*/2)(*z*−z*) for any z=x+iy∈C [22]. Note that the quaternion conjugation operator (⋅)* is also an involution and can be written in terms of *q*,*q*^*i*^,*q*^*j*^ and *q*^*k*^ as

2.9 $$q^{*}=\frac{1}{2}(-q+q^{i}+q^{j}+q^{k}).$$

### 2.2 Analytic functions in H

To arrive at the notion of analytic (regular, monogenic) function in H, recall that due to the non-commutativity of quaternion product, there are two ways to express the quotient in the definition of quaternion derivative, as shown below.

**Proposition 2.3 (Sudbery [10])**

*Let* D⊆H *be a simply connected domain of definition of the function* f:D→H. *If for any q*∈*D*

2.10 $$\lim_{h\to 0}[(\;f(q+h)-f(q))h^{-1}]$$

*exists in* H, *then necessarily f*(*q*)=*ωq*+λ *for some* ω,λ∈H. *If for any q*∈*D*

2.11 $$\lim_{h\to 0}[h^{-1}(\;f(q+h)-f(q))]$$

*exists in* H, *then necessarily f*(*q*)=*qν*+λ *for some* ν,λ∈H.

Proposition 2.3 is discussed in [10,23] and indicates that the traditional definitions of derivative in (2.10) and (2.11) are too restrictive and apply only to linear functions of quaternions. One attempt to relax this constraint is due to Fueter [24,25], whose analyticity condition is termed the Cauchy–Riemann–Fueter (CRF) equation, and is given by [10,11]

2.12 $$\begin{array}{rl} & \mathrm{Left}\;\mathrm{CRF}:\;\frac{\partial f}{\partial q_{a}}+\frac{\partial f}{\partial q_{b}}i+\frac{\partial f}{\partial q_{c}}j+\frac{\partial f}{\partial q_{d}}k=0\\ & \mathrm{Right}\;\mathrm{CRF}:\;\frac{\partial f}{\partial q_{a}}+i\frac{\partial f}{\partial q_{b}}+j\frac{\partial f}{\partial q_{c}}+k\frac{\partial f}{\partial q_{d}}=0. \end{array}\}$$

The limitations of the CRF condition were pointed out by Gentili & Struppa in [14,26], who showed that general polynomial functions (even the identity *f*(*q*)=*q*) satisfy neither the left CRF nor the right CRF. To further relax the analyticity condition, a *local* analyticity condition (LAC) was proposed in [12], by using the polar form of a quaternion, to give

2.13 $$\begin{array}{rl} & \mathrm{Left}\;\mathrm{LAC}:\;\frac{\partial f}{\partial q_{a}}+\left(q_{b}\frac{\partial f}{\partial q_{a}}+q_{c}\frac{\partial f}{\partial q_{c}}+q_{d}\frac{\partial f}{\partial q_{d}}\right)\frac{V_{q}}{|V_{q}{|}^{2}}=0\\ & \mathrm{Right}\;\mathrm{LAC}:\;\frac{\partial f}{\partial q_{a}}+\frac{V_{q}}{|V_{q}{|}^{2}}\left(q_{b}\frac{\partial f}{\partial q_{a}}+q_{c}\frac{\partial f}{\partial q_{c}}+q_{d}\frac{\partial f}{\partial q_{d}}\right)=0, \end{array}\}$$

where *q*=*q*_*a*_+*V*_*q*_ and *V*_*q*_=*iq*_*b*_+*jq*_*c*_+*kq*_*d*_. The theory of local analyticity is now well developed, and we refer the reader to [14,26,27] for the slice regular functions and to [28] for applications. More recent work in this area includes [29,30], and references therein. The advantage of the LAC is that both the polynomial functions of *q* and some elementary functions satisfy either the left LAC or the right LAC. However, in general, the products and compositions of two LAC functions *f* and *g* no longer meet the local analytic condition. For example, if *f*(*q*)=*q* and *g*(*q*)=*ωq*, ω∈H, then *f* and *g* satisfy the left LAC, but the product *fg*=*qωq* does not satisfy the left LAC. The same applies for the right LAC, when the function *g* is written as *g*(*q*)=*qω*.

Quaternion derivative is defined only for analytic functions; however, in optimization it is often required that the objective function to be minimized or maximized is real-valued. A typical example is the mean square type objective function given by

2.14 $$J(\text{w})=∥f(\text{w}){∥}^{2}.$$

Note that according to the definition of analytic (regular) function given in [10–12,14,26,27], the function *J* is not analytic. In order to take its derivatives (but not limited to only such real quadratic functions), we can, however, use the HR calculus [13] which extends the complex CR calculus [15,17,31] to the quaternion field. This generalization is not trivial, and the rules of the CR and HR calculus are different; for more detail see §3.

**Remark 2.4**

It is important to note that the left (right) terminology introduced in (2.12), (2.13) and in the sequel differs from that in [10–12,14,27]. We adopt the use of the left (right) terminology based on the position of ∂*f*/∂*q*_*a*_, ∂*f*/∂*q*_*b*_, ∂*f*/∂*q*_*c*_ and ∂*f*/∂*q*_*d*_, rather than on the positions of imaginary units *i*,*j*,*k*. Although this is only a notational difference, we later show that the left derivatives (using this convenience) in definitions 3.2 and 4.1 are in this way equipped with a left constant rule (4.3), that is, the *left* constant comes out from the *left* derivative of product, and the left derivatives stand on the left side of the quaternion differential in (A 12) and (A 16). This also allows for a consistent, intuitive and physically meaningful use of terminology.

## 3. The HR calculus

Optimization problems involving quaternions arise in a number of applications in control theory, signal processing, robotics and biomechanics. Solutions often require a first- or second-order approximation of the objective function; however, real functions of quaternion variables are essentially non-analytic. The recently proposed HR calculus [13] solves these issues through the use of quaternion involutions. The HR derivatives (the derivation of HR calculus is given in appendix A) are introduced below.

**Definition 3.1 (real-differentiability [10])**

A function *f*(*q*)=*f*_*a*_(*q*_*a*_,*q*_*b*_,*q*_*c*_,*q*_*d*_)+ *if*_*b*_(*q*_*a*_,*q*_*b*_,*q*_*c*_,*q*_*d*_)+*jf*_*c*_(*q*_*a*_,*q*_*b*_,*q*_*c*_,*q*_*d*_)+*kf*_*d*_(*q*_*a*_,*q*_*b*_,*q*_*c*_,*q*_*d*_) is called real differentiable when *f*_*a*_(*q*_*a*_,*q*_*b*_,*q*_*c*_,*q*_*d*_), *f*_*b*_(*q*_*a*_,*q*_*b*_,*q*_*c*_,*q*_*d*_), *f*_*c*_(*q*_*a*_,*q*_*b*_,*q*_*c*_,*q*_*d*_) and *f*_*d*_(*q*_*a*_,*q*_*b*_,*q*_*c*_,*q*_*d*_) are differentiable as functions of real variables *q*_*a*_,*q*_*b*_,*q*_*c*_ and *q*_*d*_.

**Definition 3.2 (the HR derivatives [13])**

If f:H→H is real-differentiable, then the formal left HR derivatives of the function *f* with respect to {*q*,*q*^*i*^,*q*^*j*^,*q*^*k*^} and {q*,*q*^i*^,*q*^j*^,*q*^k*^} are defined as

3.1 $${\left[\begin{array}{cc} \frac{\partial f}{\partial q}, & \frac{\partial f}{\partial q^{*}}\\ \frac{\partial f}{\partial q^{i}}, & \frac{\partial f}{\partial q^{i*}}\\ \frac{\partial f}{\partial q^{j}}, & \frac{\partial f}{\partial q^{j*}}\\ \frac{\partial f}{\partial q^{k}}, & \frac{\partial f}{\partial q^{k*}} \end{array}\right]}^{T}=\frac{1}{4}{\left[\begin{array}{cc} \frac{\partial f}{\partial q_{a}}, & \frac{\partial f}{\partial q_{a}}\\ \frac{\partial f}{\partial q_{b}}, & -\frac{\partial f}{\partial q_{b}}\\ \frac{\partial f}{\partial q_{c}}, & -\frac{\partial f}{\partial q_{c}}\\ \frac{\partial f}{\partial q_{d}}, & -\frac{\partial f}{\partial q_{d}} \end{array}\right]}^{T}\left[\begin{array}{cccc} 1 & 1 & 1 & 1\\ -i & -i & i & i\\ -j & j & -j & j\\ -k & k & k & -k \end{array}\right]$$

and the formal right HR derivatives of the function *f* are defined as

3.2 $$\left[\begin{array}{cc} \frac{\partial_{r}\;f}{\partial q}, & \frac{\partial_{r}\;f}{\partial q^{*}}\\ \frac{\partial_{r}\;f}{\partial q^{i}}, & \frac{\partial_{r}\;f}{\partial q^{i*}}\\ \frac{\partial_{r}\;f}{\partial q^{j}}, & \frac{\partial_{r}\;f}{\partial q^{j*}}\\ \frac{\partial_{r}\;f}{\partial q^{k}}, & \frac{\partial_{r}\;f}{\partial q^{k*}} \end{array}\right]=\frac{1}{4}\left[\begin{array}{cccc} 1 & -i & -j & -k\\ 1 & -i & j & k\\ 1 & i & -j & k\\ 1 & i & j & -k \end{array}\right]\;\left[\begin{array}{cc} \frac{\partial f}{\partial q_{a}}, & \frac{\partial f}{\partial q_{a}}\\ \frac{\partial f}{\partial q_{b}}, & -\frac{\partial f}{\partial q_{b}}\\ \frac{\partial f}{\partial q_{c}}, & -\frac{\partial f}{\partial q_{c}}\\ \frac{\partial f}{\partial q_{d}}, & -\frac{\partial f}{\partial q_{d}} \end{array}\right],$$

where ∂*f*/∂*q*_*a*_, ∂*f*/∂*q*_*b*_, ∂*f*/∂*q*_*c*_ and ∂*f*/∂*q*_*d*_ are the partial derivatives of *f* with respect to *q*_*a*_, *q*_*b*_, *q*_*c*_ and *q*_*d*_.

**Remark 3.3**

It is important to note that the right HR derivatives exist if and only if the left HR derivatives also exist. The only difference between the left HR derivatives and the right HR derivatives is in the position of the partial derivative ∂*f*/∂*q*_*a*_, ∂*f*/∂*q*_*b*_, ∂*f*/∂*q*_*c*_ and ∂*f*/∂*q*_*d*_. Within the left HR derivatives, ∂*f*/∂*q*_*a*_,∂*f*/∂*q*_*b*_,∂*f*/∂*q*_*c*_ and ∂*f*/∂*q*_*d*_ stand on the left side and imaginary units *i*,*j*,*k* on the right side (cf. (3.1)). It is exactly the opposite case for right HR derivatives. Note that the terms ∂*f*/∂*q*_*a*_,∂*f*/∂*q*_*b*_,∂*f*/∂*q*_*c*_ and ∂*f*/∂*q*_*d*_ cannot swap positions with the imaginary units *i*,*j*,*k* because of the non-commutative nature of quaternion product.

### 3.1 The validity of the traditional product rule

A straightforward use of the HR derivatives may become too tedious for complicated functions, for example, for the power function *f*(*q*)=*q*^*n*^. This is because the HR calculus does not satisfy the traditional product rule which would simplify the calculation. Indeed, for two quaternion functions *f*(*q*) and *g*(*q*), in general we have ∂( *fg*)/∂*q*≠*f*(∂*g*/∂*q*)+∂*f*/∂*qg*. We shall illustrate this difficulty through the following two examples.

**Example 3.4**

Find the HR derivative of the function f:H→H given by

3.3 $$f(q)=q^{2}=q_{a}^{2}-(q_{b}^{2}+q_{c}^{2}+q_{d}^{2})+2q_{a}(iq_{b}+jq_{c}+kq_{d}),$$

where *q*=*q*_*a*_+*iq*_*b*_+*jq*_*c*_+*kq*_*d*_, $q_{a},q_{b},q_{c},q_{d}\in R$.

**Solution**: By definition 3.2, the left HR derivative of *q*^2^ becomes

3.4 $$\begin{array}{rl} \frac{\partial (q^{2})}{\partial q} & =\frac{1}{4}\left(\frac{\partial q^{2}}{\partial q_{a}}-\frac{\partial q^{2}}{\partial q_{b}}i-\frac{\partial q^{2}}{\partial q_{c}}j-\frac{\partial q^{2}}{\partial q_{d}}k\right)\\ & =\frac{1}{4}(2q_{a}+2(iq_{b}+jq_{c}+kq_{d})-(-2q_{b}+2q_{a}i)i-(-2q_{c}+2q_{a}j)j-(-2q_{d}+2q_{a}k)k)\\ & =\frac{1}{4}(8q_{a}+4q_{b}i+4q_{c}j+4q_{d}k)=q+R(q). \end{array}$$

Alternatively, *q*(∂*q*/∂*q*)+(∂*q*/∂*q*)*q*=2*q*. This shows the traditional product rule is not valid.

**Example 3.5**

Find the HR derivative of the function f:H→H given by

3.5 $$f(q)=|q{|}^{2}=qq^{*}.$$

**Solution**: By definition 3.2, the HR derivative of |*q*|^2^ is

3.6 $$\frac{\partial (|q{|}^{2})}{\partial q}=\frac{1}{4}\left(\frac{\partial |q{|}^{2}}{\partial q_{a}}-\frac{\partial |q{|}^{2}}{\partial q_{b}}i-\frac{\partial |q{|}^{2}}{\partial q_{c}}j-\frac{\partial |q{|}^{2}}{\partial q_{d}}k\right)=\frac{1}{4}(2q_{a}-2q_{b}i-2q_{c}j-2q_{d}k)=\frac{1}{2}q^{*},$$

while *q*(∂q*/∂*q*)+(∂*q*/∂*q*)q*=−*q*/2+q*, and thus the traditional product rule is not valid.

**Remark 3.6**

Examples 3.4 and 3.5 show that the left HR derivatives do not admit the traditional product rule. Similarly, the traditional product rule is not applicable for the right HR derivative in definition 3.2.

## 4. The generalization of HR calculus: generalized HR derivatives

We now introduce the novel GHR derivatives which comprise both the product and chain rules. This is archived by replacing the basis {1,*i*,*j*,*k*} in definition 3.2 with a general orthogonal basis {1,*i*^*μ*^,*j*^*μ*^,*k*^*μ*^}, see also (2.5). The derivation of the GHR calculus is similar to that of the HR calculus in appendix A and is omitted for space considerations.

**Definition 4.1 (the generalized HR derivatives)**

If f:H→H is real-differentiable, then the left GHR derivatives of the function *f* with respect to *q*^*μ*^ and *q*^μ*^ (μ≠0,μ∈H) are defined as

4.1 $$\begin{array}{rl} \frac{\partial f}{\partial q^{\mu }} & =\frac{1}{4}\left(\frac{\partial f}{\partial q_{a}}-\frac{\partial f}{\partial q_{b}}i^{\mu }-\frac{\partial f}{\partial q_{c}}j^{\mu }-\frac{\partial f}{\partial q_{d}}k^{\mu }\right)\\ \;\text{and}\;\;\frac{\partial f}{\partial q^{\mu *}} & =\frac{1}{4}\left(\frac{\partial f}{\partial q_{a}}+\frac{\partial f}{\partial q_{b}}i^{\mu }+\frac{\partial f}{\partial q_{c}}j^{\mu }+\frac{\partial f}{\partial q_{d}}k^{\mu }\right), \end{array}\}$$

while the right GHR derivatives are defined as

4.2 $$\begin{array}{rl} \frac{\partial_{r}\;f}{\partial q^{\mu }} & =\frac{1}{4}\left(\frac{\partial f}{\partial q_{a}}-i^{\mu }\frac{\partial f}{\partial q_{b}}-j^{\mu }\frac{\partial f}{\partial q_{c}}-k^{\mu }\frac{\partial f}{\partial q_{d}}\right)\\ \;\text{and}\;\;\frac{\partial_{r}\;f}{\partial q^{\mu *}} & =\frac{1}{4}\left(\frac{\partial f}{\partial q_{a}}+i^{\mu }\frac{\partial f}{\partial q_{b}}+j^{\mu }\frac{\partial f}{\partial q_{c}}+k^{\mu }\frac{\partial f}{\partial q_{d}}\right), \end{array}\}$$

where *q*=*q*_*a*_+*iq*_*b*_+*jq*_*c*_+*kq*_*d*_, $q_{a},q_{b},q_{c},q_{d}\in R$, ∂*f*/∂*q*_*a*_, ∂*f*/∂*q*_*b*_, ∂*f*/∂*q*_*c*_ and ∂*f*/∂*q*_*d*_ are the partial derivatives of *f* with respect to *q*_*a*_, *q*_*b*_, *q*_*c*_ and *q*_*d*_, while the set {1,*i*^*μ*^,*j*^*μ*^,*k*^*μ*^} is an orthogonal basis of H.

**Remark 4.2**

The GHR derivatives are more concise and physically more intuitive than the HR derivatives, which are a special case of the GHR derivatives for *μ*={1,*i*,*j*,*k*} (definitions 3.2 and 4.1). The concept of the GHR derivative can also be applied to other orthogonal systems, such as {1,*η*,*η*′,*η*′′} in [32].

**Proposition 4.3 (constant rule)**

*Let* f:H→H *be real-differentiable. Then, the following holds*:

4.3 $$\frac{\partial (\nu f)}{\partial q^{\mu }}=\nu \frac{\partial f}{\partial q^{\mu }},\;\frac{\partial (\;f\nu )}{\partial q^{\mu }}=\frac{\partial f}{\partial q^{\nu \mu }}\nu$$

*and*

4.4 $$\frac{\partial_{r}(\;f\nu )}{\partial q^{\mu }}=\frac{\partial_{r}\;f}{\partial q^{\mu }}\nu ,\;\frac{\partial_{r}(\nu f)}{\partial q^{\mu }}=\nu \frac{\partial_{r}\;f}{\partial q^{\nu^{*}\mu }},$$

*where* μ,ν∈H *are non-zero quaternion constants*.

**Proof.**

By the definition of the left GHR derivative in (4.1), we have

4.5 $$\begin{array}{rl} \frac{\partial (\nu f)}{\partial q^{\mu }} & =\frac{1}{4}\left(\frac{\partial (\nu f)}{\partial q_{a}}-\frac{\partial (\nu f)}{\partial q_{b}}i^{\mu }-\frac{\partial (\nu f)}{\partial q_{c}}j^{\mu }-\frac{\partial (\nu f)}{\partial q_{d}}k^{\mu }\right)\\ & =\frac{1}{4}\left(\nu \frac{\partial f}{\partial q_{a}}-\nu \frac{\partial f}{\partial q_{b}}i^{\mu }-\nu \frac{\partial f}{\partial q_{c}}j^{\mu }-\nu \frac{\partial f}{\partial q_{d}}k^{\mu }\right)=\nu \frac{\partial f}{\partial q^{\mu }}. \end{array}$$

Using the equality (*q*^*μ*^)^*ν*^=*q*^*νμ*^ in (2.4), the second equality of (4.3) is proved as

4.6 $$\begin{array}{rl} \frac{\partial (\;f\nu )}{\partial q^{\mu }} & =\frac{1}{4}\left(\frac{\partial (\nu f)}{\partial q_{a}}-\frac{\partial (\nu f)}{\partial q_{b}}i^{\mu }-\frac{\partial (\nu f)}{\partial q_{c}}j^{\mu }-\frac{\partial (\nu f)}{\partial q_{d}}k^{\mu }\right)\\ & =\frac{1}{4}\left(\frac{\partial f}{\partial q_{a}}\nu -\frac{\partial f}{\partial q_{b}}\nu i^{\mu }-\frac{\partial f}{\partial q_{c}}\nu j^{\mu }-\nu \frac{\partial f}{\partial q_{d}}\nu k^{\mu }\right)\\ & =\frac{1}{4}\left(\frac{\partial f}{\partial q_{a}}-\frac{\partial f}{\partial q_{b}}i^{\nu \mu }-\frac{\partial f}{\partial q_{c}}j^{\nu \mu }-\nu \frac{\partial f}{\partial q_{d}}k^{\nu \mu }\right)\nu =\frac{\partial f}{\partial q^{\nu \mu }}\nu . \end{array}$$

Hence, (4.3) immediately follows, and (4.4) can be proved in a similar way. ▪

**Remark 4.4**

It is important to note that if a function *f* is premultiplied by a constant *η* in the first equality of (4.3), then the left GHR derivative of the product is equal to the left GHR derivative of *f* premultiplied by the constant, but not for postmultiplication. In other words, the left constant *ν* can come out from the derivative of the product; for this reason we refer to (4.1) as the left GHR derivative. The equalities in (4.4) complement those in (4.3). Thus, we refer to the derivatives in (4.2) as the right GHR derivatives, denoted by ∂_*r*_ in order to distinguish them from the left GHR derivatives in (4.1).

**Proposition 4.5 (conjugate rule)**

*Let* f:H→H *be real-differentiable. Then, the following holds*:

4.7 $$\frac{\partial f}{\partial q^{\mu }}={\left(\frac{\partial_{r}f^{*}}{\partial q^{\mu *}}\right)}^{*}\;and\;\frac{\partial f}{\partial q^{\mu *}}={\left(\frac{\partial_{r}f^{*}}{\partial q^{\mu }}\right)}^{*}.$$

**Proof.**

By the definition of the right GHR derivative in (4.2), we have

4.8 $${\left(\frac{\partial_{r}f^{*}}{\partial q^{\mu *}}\right)}^{*}=\frac{1}{4}{\left(\frac{\partial f^{*}}{\partial q_{a}}+i^{\mu }\frac{\partial f^{*}}{\partial q_{b}}+j^{\mu }\frac{\partial f^{*}}{\partial q_{c}}+k^{\mu }\frac{\partial f^{*}}{\partial q_{d}}\right)}^{*}.$$

Using the equalities (*pq*)*=q*p* and (q*)^*μ*^=(*q*^*μ*^)* in (2.4), the above equality becomes

4.9 $${\left(\frac{\partial_{r}f^{*}}{\partial q^{\mu *}}\right)}^{*}=\frac{1}{4}\left(\frac{\partial f}{\partial q_{a}}-\frac{\partial f}{\partial q_{b}}i^{\mu }-\frac{\partial f}{\partial q_{c}}j^{\mu }-\frac{\partial f}{\partial q_{d}}k^{\mu }\right)=\frac{\partial f}{\partial q^{\mu }}.$$

Hence, the first part of (4.7) follows, and the second part can be proved in a similar way. ▪

**Proposition 4.6 (rotation rule)**

*Let* f:H→H *be real-differentiable. Then the following holds*:

4.10 $${\left(\frac{\partial f}{\partial q^{\mu }}\right)}^{\nu }=\frac{\partial f^{\nu }}{\partial q^{\nu \mu }}\;and\;{\left(\frac{\partial f}{\partial q^{\mu *}}\right)}^{\nu }=\frac{\partial f^{\nu }}{\partial q^{\nu \mu *}}.$$

**Proof.**

Using the equalities in (2.4) and the left GHR derivative in (4.1), we have

4.11 $$\begin{array}{rl} {\left(\frac{\partial f}{\partial q^{\mu }}\right)}^{\nu } & =\frac{1}{4}{\left(\frac{\partial f}{\partial q_{a}}-\frac{\partial f}{\partial q_{b}}i^{\mu }-\frac{\partial f}{\partial q_{c}}j^{\mu }-\frac{\partial f}{\partial q_{d}}k^{\mu }\right)}^{\nu }\\ & =\frac{1}{4}\left(\frac{\partial f^{\nu }}{\partial q_{a}}-\frac{\partial f^{\nu }}{\partial q_{b}}i^{\nu \mu }-\frac{\partial f^{\nu }}{\partial q_{c}}j^{\nu \mu }-\frac{\partial f^{\nu }}{\partial q_{d}}k^{\nu \mu }\right)=\frac{\partial f^{\nu }}{\partial q^{\nu \mu }}. \end{array}$$

Hence, the first part of (4.10) follows, and the second part can be proved in a similar way. ▪

**Corollary 4.7**

*Let* f:H→H *be real-differentiable. Then, the following holds*:

4.12 $${\left(\frac{\partial f}{\partial q^{\eta }}\right)}^{\eta }=\frac{\partial f^{\eta }}{\partial q}\;and\;{\left(\frac{\partial f}{\partial q^{\eta *}}\right)}^{\eta }=\frac{\partial f^{\eta }}{\partial q^{*}},\;\forall \;\eta \in \{1,i,j,k\}.$$

**Proof.**

Since ∂*f*/∂*q*^*ηη*^=∂*f*/∂*q* for any *η*∈{1,*i*,*j*,*k*}, then the proof follows directly from proposition 4.6. ▪

**Proposition 4.8**

*Let* f:H→R *be real-differentiable. Then, the following holds*:

4.13 $$\frac{\partial f}{\partial q^{\mu }}={\left(\frac{\partial f}{\partial q^{\mu *}}\right)}^{*}=\frac{\partial_{r}\;f}{\partial q^{\mu }}\;and\;\frac{\partial f}{\partial q^{\mu *}}={\left(\frac{\partial f}{\partial q^{\mu }}\right)}^{*}=\frac{\partial_{r}\;f}{\partial q^{\mu *}}.$$

**Proof.**

Since *f* is real-valued, its partial derivatives ∂*f*/∂*q*_*a*_, ∂*f*/∂*q*_*b*_, ∂*f*/∂*q*_*c*_ and ∂*f*/∂*q*_*d*_ are real numbers, which yields (∂*f*/∂*ξ*)*=∂*f*/∂*ξ*, where *ξ*∈{*q*_*a*_,*q*_*b*_,*q*_*c*_,*q*_*d*_}. Using the equality (*q*^*μ*^)*=(q*)^*μ*^ in (2.4) and the left GHR derivative in (4.1), we have

4.14 $$\begin{array}{rl} {\left(\frac{\partial f}{\partial q^{\mu *}}\right)}^{*} & =\frac{1}{4}{\left(\frac{\partial f}{\partial q_{a}}+\frac{\partial f}{\partial q_{b}}i^{\mu }+\frac{\partial f}{\partial q_{c}}j^{\mu }+\frac{\partial f}{\partial q_{d}}k^{\mu }\right)}^{*}\\ & =\frac{1}{4}\left(\frac{\partial f}{\partial q_{a}}-i^{\mu }\frac{\partial f}{\partial q_{b}}-j^{\mu }\frac{\partial f}{\partial q_{c}}-k^{\mu }\frac{\partial f}{\partial q_{d}}\right)=\frac{\partial_{r}\;f}{\partial q^{\mu }}\\ & =\frac{1}{4}\left(\frac{\partial f}{\partial q_{a}}-\frac{\partial f}{\partial q_{b}}i^{\mu }-\frac{\partial f}{\partial q_{c}}j^{\mu }-\frac{\partial f}{\partial q_{d}}k^{\mu }\right)=\frac{\partial f}{\partial q^{\mu }}. \end{array}$$

Hence, the first part of (4.13) follows, and the second part can be proved in a similar way. ▪

**Remark 4.9**

From the identity (4.13), observe that the left GHR derivative is equal to the right GHR derivative if the function *f* is real-valued. This result is instrumental for practical applications of quaternion optimization, where the objective function (or cost function) is frequently real-valued. By virtue of the GHR calculus, the choice of the left/right GHR derivative therefore becomes obsolete as shown in (4.13). In the sequel, without loss in generality we shall mainly focus on the left GHR derivatives.

### 4.1 The novel product rule

We now introduce a novel product rule into quaternion analysis and show that the traditional product rule is a special case of the proposed product rule in corollary 4.11.

**Theorem 4.10 (product rule of left GHR)**

*If the functions* f,g:H→H *are real-differentiable, then so too is their product fg, so that
*

4.15 $$\frac{\partial (\;fg)}{\partial q^{\mu }}=f\frac{\partial g}{\partial q^{\mu }}+\frac{\partial f}{\partial q^{g\mu }}g\;and\;\frac{\partial (\;fg)}{\partial q^{\mu *}}=f\frac{\partial g}{\partial q^{\mu *}}+\frac{\partial f}{\partial q^{g\mu *}}g,$$

*where ∂f/∂q*^*gμ*^ *and ∂f/∂q*^gμ*^ *can be obtained by replacing μ with gμ in definition 4.1.*

**Proof.**

The proof is given in appendix B. ▪

**Corollary 4.11**

*If the functions* f:H→H *and* g:H→R *are real-differentiable, then their product fg satisfies the traditional product rule*

4.16 $$\frac{\partial (\;fg)}{\partial q^{\mu }}=f\frac{\partial g}{\partial q^{\mu }}+\frac{\partial f}{\partial q^{\mu }}g\;and\;\frac{\partial (\;fg)}{\partial q^{\mu *}}=f\frac{\partial g}{\partial q^{\mu *}}+\frac{\partial f}{\partial q^{\mu *}}g,$$

*where* ∂*f*/∂*q*^*μ*^ *and* ∂*f*/∂*q*^μ*^ *are the left GHR derivatives in definition 4.1*.

**Proof.**

Since *q*^*gμ*^=*q*^*μ*^ and *q*^gμ*^=*q*^μ*^ for a real function g:H→R, the corollary follows. ▪

We now present some GHR derivatives of nonlinear quaternion functions enabled by the GHR calculus. These are very useful in applications, such as in nonlinear adaptive filters and quaternion-valued neural networks.

**Example 4.12 (split-quaternion function)**

Find the GHR derivative of a split-quaternion function f:H→H given by

4.17 $$f(q)=\varphi (q_{a})+j\varphi (q_{b})+j\varphi (q_{c})+k\varphi (q_{d}),$$

where $q=q_{a}+iq_{b}+jq_{c}+kq_{d}\in R$ and φ:R→R is a real-valued differentiable function.

**Solution**: By the definition of the left GHR derivatives in (4.1), it follows that

4.18 $$\begin{array}{rl} \frac{\partial f(q)}{\partial q} & =\frac{1}{4}\left(\frac{\partial f(q)}{\partial q_{a}}-\frac{\partial f(q)}{\partial q_{a}}i-\frac{\partial f(q)}{\partial q_{a}}j-\frac{\partial f(q)}{\partial q_{a}}k\right)\\ & =\frac{1}{4}(\varphi^{\prime }(q_{a})+\varphi^{\prime }(q_{b})+\varphi^{\prime }(q_{c})+\varphi^{\prime }(q_{d})) \end{array}$$

and

4.19 $$\begin{array}{rl} \frac{\partial f(q)}{\partial q^{*}} & =\frac{1}{4}\left(\frac{\partial f(q)}{\partial q_{a}}+\frac{\partial f(q)}{\partial q_{a}}i+\frac{\partial f(q)}{\partial q_{a}}j+\frac{\partial f(q)}{\partial q_{a}}k\right)\\ & =\frac{1}{4}(\varphi^{\prime }(q_{a})-\varphi^{\prime }(q_{b})-\varphi^{\prime }(q_{c})-\varphi^{\prime }(q_{d})). \end{array}$$

This shows that the GHR derivatives of the split-quaternion function are real-valued.

**Example 4.13 (power function)**

Find the GHR derivative of the power function f:H→H given by

4.20 $$f(q)=q^{n},$$

where *n* is any positive integer.

**Solution**: Using the product rule in theorem 4.10, it follows that

4.21 $$\frac{\partial q^{n}}{\partial q^{\mu }}\mu =\frac{\partial (qq^{n-1})}{\partial q^{\mu }}\mu =q\frac{\partial q^{n-1}}{\partial q^{\mu }}\mu +\frac{\partial q}{\partial q^{q^{n-1}\mu }}q^{n-1}\mu =q\frac{\partial q^{n-1}}{\partial q^{\mu }}\mu +R(q^{n-1}\mu ),$$

where the term (∂*q*/∂*q*^*μ*^)*μ*, given in table 1, was used in the last equality. Note that the above expression is recurrent in (∂*q*^*n*^/∂*q*^*μ*^)*μ*. Upon expanding this expression and using the initial condition $\left(\partial q/\partial q^{\mu })\mu =R(\mu \right)$, this yields

4.22 $$\frac{\partial q^{n}}{\partial q^{\mu }}\mu =\sum_{m=1}^{n}q^{n-m}R(q^{m-1}\mu ).$$

In a similar manner, we have

4.23 $$\frac{\partial q^{n}}{\partial q^{\mu *}}\mu =\frac{\partial (qq^{n-1})}{\partial q^{\mu *}}\mu =q\frac{\partial q^{n-1}}{\partial q^{\mu *}}\mu +\frac{\partial q}{\partial q^{q^{n-1}\mu *}}q^{n-1}\mu =q\frac{\partial q^{n-1}}{\partial q^{\mu *}}\mu -\frac{1}{2}{\left(q^{n-1}\mu \right)}^{*},$$

which is equivalent to

4.24 $$\frac{\partial q^{n}}{\partial q^{\mu *}}\mu =-\frac{1}{2}\sum_{m=1}^{n}q^{n-m}{\left(q^{m-1}\mu \right)}^{*}.$$

**Table 1.** Summary of the rules for the left GHR derivatives.

*f*(*q*)	$\frac{\partial f}{\partial q^{\mu }}\mu$	$\frac{\partial f}{\partial q^{\mu *}}\mu$	note
*q*	R(μ)	$-\frac{1}{2}\mu^{*}$	—
*ωq*	ωR(μ)	$-\frac{1}{2}\omega \mu^{*}$	∀ ω∈H
*qν*	R(νμ)	$-\frac{1}{2}{\left(\nu \mu \right)}^{*}$	∀ ν∈H
*ωqν*+λ	ωR(νμ)	$-\frac{1}{2}\omega {\left(\nu \mu \right)}^{*}$	∀ ω,ν,λ∈H
q*	$-\frac{1}{2}\mu^{*}$	R(μ)	—
ωq*	$-\frac{1}{2}\omega \mu^{*}$	ωR(μ)	∀ ω∈H
q*ν	$-\frac{1}{2}{\left(\nu \mu \right)}^{*}$	R(νμ)	∀ ν∈H
ωq*ν+λ	$-\frac{1}{2}\omega {\left(\nu \mu \right)}^{*}$	ωR(νμ)	∀ ω,ν,λ∈H
*q*^−1^	$-q^{-1}R(q^{-1}\mu )$	$\frac{1}{2}q^{-1}\mu^{*}{\left(q^{*}\right)}^{-1}$	—
(q*)^−1^	$\frac{1}{2}{\left(q^{*}\right)}^{-1}\mu^{*}q^{-1}$	$-{\left(q^{*}\right)}^{-1}R({\left(q^{*}\right)}^{-1}\mu )$	—
(*ωqν*+λ)^−1^	−fωR(νfμ)	$\frac{1}{2}f\omega {\left(\nu f\mu \right)}^{*}$	∀ ω,ν,λ∈H
(ωq*ν+λ)^−1^	$\frac{1}{2}f\omega {\left(\nu f\mu \right)}^{*}$	−fωR(νfμ)	∀ ω,ν,λ∈H
*q*^2^	qR(μ)+R(qμ)	$-\frac{1}{2}q\mu^{*}-\frac{1}{2}{\left(q\mu \right)}^{*}$	—
(q*)^2^	$-\frac{1}{2}q^{*}\mu^{*}-\frac{1}{2}{\left(q^{*}\mu \right)}^{*}$	$q^{*}R(\mu )+R(q^{*}\mu )$	—
(*ωqν*+λ)^2^	gωR(νμ)+ωR(νgμ)	$-\frac{1}{2}g\omega {\left(\nu \mu \right)}^{*}-\frac{1}{2}\omega {\left(\nu g\mu \right)}^{*}$	*g*=*ωqν*+λ
(ωq*ν+λ)^2^	$-\frac{1}{2}g\omega {\left(\nu \mu \right)}^{*}-\frac{1}{2}\omega {\left(\nu g\mu \right)}^{*}$	gωR(νμ)+ωR(νgμ)	*g*=ωq*ν+λ
R(q)	$\frac{1}{4}\mu$	$\frac{1}{4}\mu$	—
R(ωqν+λ)	$\frac{1}{4}\mu \nu \omega$	$\frac{1}{4}\mu \omega^{*}\nu^{*}$	∀ ω,ν,λ∈H
$R(\omega q^{*}\nu +\lambda )$	$\frac{1}{4}\mu \omega^{*}\nu^{*}$	$\frac{1}{4}\mu \nu \omega$	∀ ω,ν,λ∈H
|*q*|	$\frac{1}{4|q|}\mu q^{*}$	$\frac{1}{4|q|}\mu q$	—
|*q*|^2^	$\frac{1}{2}\mu q^{*}$	$\frac{1}{2}\mu q$	—
|*ωqν*+λ|	$\frac{1}{4|g|}\mu \nu g^{*}\omega$	$\frac{1}{4|g|}\mu \omega^{*}g\nu^{*}$	*g*=*ωqν*+λ
|ωq*ν+λ|	$\frac{1}{4|g|}\mu \omega^{*}g\nu^{*}$	$\frac{1}{4|g|}\mu \nu g^{*}\omega$	*g*=ωq*ν+λ
|*ωqν*+λ|^2^	$\frac{1}{2}\mu \nu g^{*}\omega$	$\frac{1}{2}\mu \omega^{*}g\nu^{*}$	*g*=*ωqν*+λ
|ωq*ν+λ|^2^	$\frac{1}{2}\mu \omega^{*}g\nu^{*}$	$\frac{1}{2}\mu \nu g^{*}\omega$	*g*=ωq*ν+λ
$\frac{q}{|q|}$	$\frac{1}{|q|}R(\mu )-\frac{1}{4|q{|}^{3}}q\mu q^{*}$	$-\frac{1}{2|q|}\mu^{*}-\frac{1}{4|q{|}^{3}}q\mu q$	—
$\frac{q^{*}}{|q|}$	$-\frac{1}{2|q|}\mu^{*}-\frac{1}{4|q{|}^{3}}q^{*}\mu q^{*}$	$\frac{1}{|q|}R(\mu )-\frac{1}{4|q{|}^{3}}q^{*}\mu q$	—
$\frac{\omega q\nu +\lambda }{|\omega q\nu +\lambda |}$	$\frac{\omega }{2|g|}R(\nu \mu )+\frac{g}{4|g{|}^{3}}\nu^{*}{\left(\omega^{*}g\mu \right)}^{*}$	$-\frac{\omega }{4|g|}{\left(\nu \mu \right)}^{*}-\frac{g}{2|g{|}^{3}}\nu^{*}R(\omega^{*}g\mu )$	*g*=*ωqν*+λ
$\frac{\omega q^{*}\nu +\lambda }{|\omega q^{*}\nu +\lambda |}$	$-\frac{\omega }{2|g|}{\left(\nu \mu \right)}^{*}-\frac{f}{|g|}\frac{\partial |g|}{\partial q^{\mu }}\mu$	$\frac{\omega }{|g|}R(\nu \mu )-\frac{f}{|g|}\frac{\partial |g|}{\partial q^{\mu *}}\mu$	*g*=ωq*ν+λ

**Example 4.14 (exponential function)(power function)**

Find the GHR derivative of the function f:H→H given by

4.25 $$\exp(q)≜\sum_{n=0}^{+\infty }\frac{q^{n}}{n!}.$$

**Solution**: From (4.22), it follows that

4.26 $$\frac{\partial \exp(q)}{\partial q^{\mu }}\mu =\sum_{n=0}^{+\infty }\frac{1}{n!}\sum_{m=1}^{n}q^{n-m}R(q^{m-1}\mu ).$$

In a similar manner, we have

4.27 $$\frac{\partial \exp(q)}{\partial q^{\mu *}}\mu =-\frac{1}{2}\sum_{n=0}^{+\infty }\frac{1}{n!}\sum_{m=1}^{n}q^{n-m}{\left(q^{m-1}\mu \right)}^{*}.$$

**Remark 4.15**

The exponential function is the most important elementary function, as both trigonometric functions and hyperbolic functions can be expressed in terms of the exponential function. The elementary function in example 4.14 is a power series, and it does not change the direction of the vector part of quaternion. Therefore, such elementary functions can swap positions with a quaternion *q*, i.e. *f*(*q*)*q*=*qf*(*q*), giving an important property, f*(*q*)=*f*(q*), which can be used in practical applications, such as quaternion neural networks [33] and quaternion nonlinear adaptive filters [28]. It is important to note that if a quaternion variable *q* degenerates into a real variable *x* in the definitions of elementary functions in this subsection, then the GHR derivatives simplify into the standard real derivatives, e.g. the GHR derivative of the power function in (4.22) will become *nx*^*n*−1^. Therefore, the GHR derivatives are a generalized form of the real derivatives and the real derivatives are a special case of the GHR derivatives.

### 4.2 The chain rule

Another advantage of the GHR derivatives is that they admit the chain rule, which is formulated in the following theorem.

**Theorem 4.16 (chain rule of left GHR)**

*Let* S⊆H *and let* g:S→H *be real-differentiable at an interior point q of the set S. Let* T⊆H *be such that g*(*q*)∈*T for all q*∈*S. Assume that* f:T→H *is real-differentiable at an interior point g*(*q*)∈*T, then the composite function f*(*g*(*q*)) *satisfies the following chain rules*:

4.28 $$\begin{array}{rl} \frac{\partial f(g(q))}{\partial q^{\mu }} & =\frac{\partial f}{\partial g^{\nu }}\frac{\partial g^{\nu }}{\partial q^{\mu }}+\frac{\partial f}{\partial g^{\nu i}}\frac{\partial g^{\nu i}}{\partial q^{\mu }}+\frac{\partial f}{\partial g^{\nu j}}\frac{\partial g^{\nu j}}{\partial q^{\mu }}+\frac{\partial f}{\partial g^{\nu k}}\frac{\partial g^{\nu k}}{\partial q^{\mu }}\\ \frac{\partial f(g(q))}{\partial q^{\mu *}} & =\frac{\partial f}{\partial g^{\nu }}\frac{\partial g^{\nu }}{\partial q^{\mu *}}+\frac{\partial f}{\partial g^{\nu i}}\frac{\partial g^{\nu i}}{\partial q^{\mu *}}+\frac{\partial f}{\partial g^{\nu j}}\frac{\partial g^{\nu j}}{\partial q^{\mu *}}+\frac{\partial f}{\partial g^{\nu k}}\frac{\partial g^{\nu k}}{\partial q^{\mu *}} \end{array}\}$$

*and*

4.29 $$\begin{array}{rl} \frac{\partial f(g(q))}{\partial q^{\mu }} & =\frac{\partial f}{\partial g^{\nu *}}\frac{\partial g^{\nu *}}{\partial q^{\mu }}+\frac{\partial f}{\partial g^{\nu i*}}\frac{\partial g^{\nu i*}}{\partial q^{\mu }}+\frac{\partial f}{\partial g^{\nu j*}}\frac{\partial g^{\nu j*}}{\partial q^{\mu }}+\frac{\partial f}{\partial g^{\nu k*}}\frac{\partial g^{\nu k*}}{\partial q^{\mu }}\\ \frac{\partial f(g(q))}{\partial q^{\mu *}} & =\frac{\partial f}{\partial g^{\nu *}}\frac{\partial g^{\nu *}}{\partial q^{\mu *}}+\frac{\partial f}{\partial g^{\nu i*}}\frac{\partial g^{\nu i*}}{\partial q^{\mu *}}+\frac{\partial f}{\partial g^{\nu j*}}\frac{\partial g^{\nu j*}}{\partial q^{\mu *}}+\frac{\partial f}{\partial g^{\nu k*}}\frac{\partial g^{\nu k*}}{\partial q^{\mu *}}, \end{array}\}$$

*where* μ,ν∈H,μν≠0, *and* ∂*f*/∂*g*^*ν*^=∂*f*(*g*)/∂*g*^*ν*^ *and* ∂*f*/∂*g*^ν*^=∂*f*(*g*)/∂*g*^ν*^ *are the left GHR derivatives.*

**Proof.**

The proof of theorem 4.16 is given in appendix C. ▪

Theorem 4.16 is also valid for complex-valued and real-valued composite functions of quaternion variables, as stated in the following two corollaries, the proofs of which are similar to that of theorem 4.16, and are thus omitted.

**Corollary 4.17**

*Let* S⊆H *and let* g:S→C *be real-differentiable at an interior point q of the set S. Let* T⊆C *be such that g*(*q*)∈*T for all q*∈*S. Assume that* f:T→C *is real-differentiable at an interior point g*(*q*)∈*T, then the left GHR derivatives of the composite function f*(*g*(*q*)) *are as follows*:

4.30 $$\frac{\partial f(g(q))}{\partial q^{\mu }}=\frac{\partial f}{\partial g}\frac{\partial g}{\partial q^{\mu }}+\frac{\partial f}{\partial g^{*}}\frac{\partial g^{*}}{\partial q^{\mu }}\;and\;\frac{\partial f(g(q))}{\partial q^{\mu *}}=\frac{\partial f}{\partial g}\frac{\partial g}{\partial q^{\mu *}}+\frac{\partial f}{\partial g^{*}}\frac{\partial g^{*}}{\partial q^{\mu *}},$$

*where* μ∈H,μ≠0, *and* ∂*f*/∂*g*=∂*f*(*g*)/∂*g and* ∂*f*/∂g*=∂*f*(*g*)/∂g* *are the complex CR derivatives within the CR calculus*.

**Corollary 4.18**

*Let* S⊆H *and let* g:S→R *be real-differentiable at an interior point q of the set S. Let* T⊆R *be such that g*(*q*)∈*T for all q*∈*S. Assume that* f:T→R *is real-differentiable at an interior point f*(*q*)∈*T, then the left GHR derivatives of the composite function f*(*g*(*q*)) *are as follows*:

4.31 $$\frac{\partial f(g(q))}{\partial q^{\mu }}=f^{\prime }(g)\frac{\partial g}{\partial q^{\mu }}\;and\;\frac{\partial f(g(q))}{\partial q^{\mu *}}=f^{\prime }(g)\frac{\partial g}{\partial q^{\mu *}},$$

*where* μ∈H,μ≠0 *and f*′(*g*) *is the real derivative of a real-valued function*.

**Theorem 4.19 (chain rule of right GHR)**

*Let* S⊆H *and let* g:S→H *be real-differentiable at an interior point q of the set S. Let* T⊆H *be such that g*(*q*)∈*T for all q*∈*S. Assume that* f:T→H *is real-differentiable at an inner point g*(*q*)∈*T, then the right GHR derivatives of the composite function f*(*g*(*q*)) *are as follows*:

4.32 $$\begin{array}{rl} \frac{\partial_{r}\;f(g(q))}{\partial q^{\mu }} & =\frac{\partial_{r}g^{\nu }}{\partial q^{\mu }}\frac{\partial_{r}\;f}{\partial g^{\nu }}+\frac{\partial_{r}g^{\nu i}}{\partial q^{\mu }}\frac{\partial_{r}\;f}{\partial g^{\nu i}}+\frac{\partial_{r}g^{\nu j}}{\partial q^{\mu }}\frac{\partial_{r}\;f}{\partial g^{\nu j}}+\frac{\partial_{r}g^{\nu k}}{\partial q^{\mu }}\frac{\partial_{r}\;f}{\partial g^{\nu k}}\\ \frac{\partial_{r}\;f(g(q))}{\partial q^{\mu *}} & =\frac{\partial_{r}g^{\nu }}{\partial q^{\mu *}}\frac{\partial_{r}\;f}{\partial g^{\nu }}+\frac{\partial_{r}g^{\nu i}}{\partial q^{\mu *}}\frac{\partial_{r}\;f}{\partial g^{\nu i}}+\frac{\partial_{r}g^{\nu j}}{\partial q^{\mu *}}\frac{\partial_{r}\;f}{\partial g^{\nu j}}+\frac{\partial_{r}g^{\nu k}}{\partial q^{\mu *}}\frac{\partial_{r}\;f}{\partial g^{\nu k}} \end{array}\}$$

*and*

4.33 $$\begin{array}{rl} \frac{\partial_{r}\;f(g(q))}{\partial q^{\mu }} & =\frac{\partial_{r}g^{\nu *}}{\partial q^{\mu }}\frac{\partial_{r}\;f}{\partial g^{\nu *}}+\frac{\partial_{r}g^{\nu i*}}{\partial q^{\mu }}\frac{\partial_{r}\;f}{\partial g^{\nu i*}}+\frac{\partial_{r}g^{\nu j*}}{\partial q^{\mu }}\frac{\partial_{r}\;f}{\partial g^{\nu j*}}+\frac{\partial_{r}g^{\nu k*}}{\partial q^{\mu }}\frac{\partial_{r}\;f}{\partial g^{\nu k*}}\\ \frac{\partial_{r}\;f(g(q))}{\partial q^{\mu *}} & =\frac{\partial_{r}g^{\nu *}}{\partial q^{\mu *}}\frac{\partial_{r}\;f}{\partial g^{\nu *}}+\frac{\partial_{r}g^{\nu i*}}{\partial q^{\mu *}}\frac{\partial_{r}\;f}{\partial g^{\nu i*}}+\frac{\partial_{r}g^{\nu j*}}{\partial q^{\mu *}}\frac{\partial_{r}\;f}{\partial g^{\nu j*}}+\frac{\partial_{r}g^{\nu k*}}{\partial q^{\mu *}}\frac{\partial_{r}\;f}{\partial g^{\nu k*}}, \end{array}\}$$

*where* μ,ν∈H,μν≠0, *and* ∂_*r*_*f*/∂*g*^*ν*^=∂_*r*_*f*(*g*)/∂*g*^*ν*^ *and* ∂_*r*_*f*/∂*g*^ν*^=∂_*r*_*f*(*g*)/∂*g*^ν*^ *are the right GHR derivatives.*

**Proof.**

The proof of theorem 4.19 is similar to that of theorem 4.16 and is thus omitted. ▪

### 4.3 Mean value theorem

The mean value theorem is one of the most important tools in calculus, and we next introduce its compact version for general quaternion-valued functions of quaternion variables.

**Theorem 4.20 (mean value theorem of left kind)**

*Consider a continuous function* f:S⊆H→H *for which the left GHR derivatives exist and are continuous in the set S. Then, for any q*_0_*,q*_1_∈*S for which the segment joining them also lies in S, we have*

4.34 $$f(q_{1})-f(q_{0})=\sum_{\mu }\int_{0}^{1}\frac{\partial f(q_{0}+t\lambda )}{\partial q^{\mu }}\lambda^{\mu }\;dt=\sum_{\mu }\int_{0}^{1}\frac{\partial f(q_{0}+t\lambda )}{\partial q^{\mu *}}\lambda^{\mu *}\;dt,$$

*where μ*∈{1,*i,j,k*}, λ=*q*_1_−*q*_0_, *and* ∂*f*(*q*_0_+*t*λ)/∂*q*^*μ*^=∂*f*(*q*)/∂*q*^*μ*^|_*q*=*q*_0_+*t*λ_ *is the left GHR derivative as in definition 4.1*.

**Proof.**

Denote *F*(*t*)=*f*(*g*(*t*)), where *g*(*t*)=*q*_0_+*t*λ and 0≤*t*≤1. Then *F*(*t*) is continuous on [0,1] and has derivatives in (0,1). By theorem 4.16, the derivative of *F*(*t*) is

4.35 $$\begin{array}{rl} F^{\prime }(t) & =\frac{\partial f(g)}{\partial g}\lambda +\frac{\partial f(g)}{\partial g^{i}}\lambda^{i}+\frac{\partial f(g)}{\partial g^{j}}\lambda^{j}+\frac{\partial f(g)}{\partial g^{k}}\lambda^{k}\\ & ={\frac{\partial f(q)}{\partial q}|}_{q=q_{0}+t\lambda }\lambda +{\frac{\partial f(q)}{\partial q^{i}}|}_{q=q_{0}+t\lambda }\lambda^{i}+{\frac{\partial f(q)}{\partial q^{j}}|}_{q=q_{0}+t\lambda }\lambda^{j}+{\frac{\partial f(q)}{\partial q^{k}}|}_{q=q_{0}+t\lambda }\lambda^{k}\\ & =\frac{\partial f(q_{0}+t\lambda )}{\partial q}\lambda +\frac{\partial f(q_{0}+t\lambda )}{\partial q^{i}}\lambda^{i}+\frac{\partial f(q_{0}+t\lambda )}{\partial q^{j}}\lambda^{j}+\frac{\partial f(q_{0}+t\lambda )}{\partial q^{k}}\lambda^{k}. \end{array}$$

Upon substituting (4.35) into $F(1)-F(0)=\int_{0}^{1}F^{\prime }(t)\;dt$, with *F*(0)=*f*(*q*_0_) and *F*(1)=*f*(*q*_1_), the theorem follows. The second equality can be proved in a similar manner. ▪

**Corollary 4.21**

*Consider a continuous function* f:S⊆H→R *for which the left GHR derivatives exist and are continuous in the set S. Then, for any q*_0_,*q*_1_∈*S for which the segment joining them also lies in S, we have*

4.36 $$f(q_{1})-f(q_{0})=4\int_{0}^{1}R\left(\frac{\partial f(q_{0}+t\lambda )}{\partial q}\lambda \right)dt=4\int_{0}^{1}R\left(\frac{\partial f(q_{0}+t\lambda )}{\partial q^{*}}\lambda^{*}\right)dt,$$

*where* λ=*q*_1_−*q*_0_ *and* ∂*f*(*q*_0_+*t*λ)/∂*q*^*μ*^=∂*f*(*q*)/∂*q*^*μ*^|_*q*=*q*_0_+*t*λ_ *is the left GHR derivative as in definition 4.1*.

**Proof.**

Function *f* is real-valued, and therefore ∂*f*/∂*q*=∂*f*^*η*^/∂*q*, where *η*∈{1,*i*,*j*,*k*}. From (2.3) and (4.10), we now have

4.37 $$\frac{\partial f}{\partial q^{\eta }}\lambda^{\eta }=\frac{\partial f^{\eta }}{\partial q^{\eta }}\lambda^{\eta }={\left(\frac{\partial f}{\partial q}\lambda \right)}^{\eta }.$$

The corollary then follows from (2.7) and theorem 4.20, while the second equality can be derived using $R(pq)=R(p^{*}q^{*})$. ▪

For λ which is sufficiently small in the modulus, the right-hand side of (4.34) can be approximated as

4.38 $$f(q_{1})-f(q_{0})\approx \sum_{\mu \in \{1,i,j,k\}}\frac{\partial f(q_{0})}{\partial q^{\mu }}\lambda^{\mu }.$$

If the left GHR derivatives of *f* are Lipschitz continuous in the vicinity of *q* and *q*_1_, with the Lipschitz constant *L*, we can estimate the error in this approximation as

4.39 $$\begin{array}{rl} & \left|f(q_{1})-f(q_{0})-\sum_{\mu \in \{1,i,j,k\}}\frac{\partial f(q_{0})}{\partial q^{\mu }}\lambda^{\mu }\right|\\ & \;\leq \sum_{\mu \in \{1,i,j,k\}}\left|\int_{0}^{1}\left(\frac{\partial f(q_{0}+t\lambda )}{\partial q^{\mu }}-\frac{\partial f(q_{0})}{\partial q^{\mu }}\right)\lambda^{\mu }\;dt\right|\leq 4\int_{0}^{1}Lt|\lambda {|}^{2}\;dt=2L|\lambda {|}^{2}. \end{array}$$

### 4.4 Taylor's theorem

We can now introduce a novel, rigorous version of Taylor's theorem for quaternion-valued functions of quaternion variables, as a generalization of the standard univariate Taylor's theorem.

**Lemma 4.22 (Apostol [34])**

*Consider a* (*k*+1)-*times continuously differentiable function* f:D⊆R→R. *If x*∈*D, then*

4.40 $$f(x_{0}+h)=f(x_{0})+f^{\prime }(x_{0})h+f^{\prime \prime }(x_{0})\frac{h^{2}}{2}+\cdots +f^{\left(k\right)}(x_{0})\frac{h^{k}}{k!}+R_{k},$$

*where the remainder R*_*k*_ *is given by*

4.41 $$R_{k}=\int_{x_{0}}^{x_{0}+h}\frac{f^{\left(k+1\right)}(t)}{k!}{\left(x_{0}+h-t\right)}^{k}\;dt.$$

**Theorem 4.23 (Taylor's theorem of left kind)**

*Consider a third-order continuous real-differentiable function* f:S⊆H→H*. If q*_0_*,q*_0_+λ∈*S such that the segment joining them also lies in S, then*

4.42 $$\begin{array}{rl} f(q_{0}+\lambda )-f(q_{0}) & =\sum_{\mu }\frac{\partial f(q_{0})}{\partial q^{\mu }}\lambda^{\mu }+\frac{1}{2}\sum_{\mu ,\nu }\frac{\partial^{2}f(q_{0})}{\partial q^{\nu }\partial q^{\mu }}\lambda^{\nu }\lambda^{\mu }+O(\lambda^{3})\\ & =\sum_{\mu }\frac{\partial f(q_{0})}{\partial q^{\mu *}}\lambda^{\mu *}+\frac{1}{2}\sum_{\mu ,\nu }\frac{\partial^{2}f(q_{0})}{\partial q^{\nu *}\partial q^{\mu *}}\lambda^{\nu *}\lambda^{\mu *}+O(\lambda^{3})\;\mathrm{as}\;\lambda \to 0, \end{array}$$

*where μ,ν*∈{1,*i,j,k*}, *and* ∂^2^*f*/∂*q*^*ν*^∂*q*^*μ*^ *and* ∂^2^*f*/∂*q*^ν*^∂*q*^μ*^ *are the second-order left GHR derivatives.*

**Proof.**

Define an auxiliary function *g*(*t*)=*f*(*q*_0_+*t*λ) with 0≤*t*≤1. Using the chain rule in theorem 4.16, we obtain

4.43 $$\begin{array}{rl} g^{\prime }(t) & =\sum_{\mu }\frac{\partial f(q_{0}+t\lambda )}{\partial q^{\mu }}\lambda^{\mu }\\ \;\text{and}\;\;g^{\prime \prime }(t) & =\sum_{\mu ,\nu }\frac{\partial^{2}f(q_{0}+t\lambda )}{\partial q^{\nu }\partial q^{\mu }}\lambda^{\nu }\lambda^{\mu },\;g^{\prime \prime \prime }(t)=\sum_{\mu ,\nu ,\eta }\frac{\partial^{3}f(q_{0}+t\lambda )}{\partial q^{\eta }\partial q^{\nu }\partial q^{\mu }}\lambda^{\eta }\lambda^{\nu }\lambda^{\mu }, \end{array}\}$$

where *μ*,*ν*,*η*∈{1,*i*,*j*,*k*}. The second-order Taylor polynomial in lemma 4.22 then gives

4.44 $$g(1)=g(0)+g^{\prime }(0)+\frac{1}{2}g^{\prime \prime }(0)+R_{2}$$

which is equivalent to

4.45 $$f(q_{0}+\lambda )=f(q_{0})+\sum_{\mu }\frac{\partial f(q_{0})}{\partial q^{\mu }}\lambda^{\mu }+\frac{1}{2}\sum_{\mu ,\nu }\frac{\partial^{2}f(q_{0})}{\partial q^{\nu }\partial q^{\mu }}\lambda^{\nu }\lambda^{\mu }+R_{2},$$

where

4.46 $$R_{2}=\int_{0}^{1}\frac{{\left(1-t\right)}^{2}}{2}g^{\prime \prime \prime }(t)\;dt=\int_{0}^{1}\frac{{\left(1-t\right)}^{2}}{2}\sum_{\mu ,\nu ,\eta }\frac{\partial^{3}f(q_{0}+t\lambda )}{\partial q^{\eta }\partial q^{\nu }\partial q^{\mu }}\lambda^{\eta }\lambda^{\nu }\lambda^{\mu }\;dt.$$

This integral contains three factors of λ, while the remaining factors are bounded. Therefore, *R*_2_ is of the order |λ|^3^, making the fraction |*R*_2_|/|λ|^3^ bounded as λ→0. Hence, the first equality of the theorem follows, and the second equality can be proved in the same way. ▪

**Corollary 4.24**

*Consider a third-order continuous real-differentiable function* f:S⊆H→R. *If q*_0_,*q*_0_+λ∈*S such that the segment joining them also lies in S, then*

4.47 $$\begin{array}{rl} f(q_{0}+\lambda )-f(q_{0}) & =4R\left(\frac{\partial f(q_{0})}{\partial q}\lambda \right)+2\sum_{\nu }R\left(\frac{\partial^{2}f(q_{0})}{\partial q^{\nu }\partial q}\lambda^{\nu }\lambda \right)+O(|\lambda {|}^{3})\\ & =4R\left(\frac{\partial f(q_{0})}{\partial q^{*}}\lambda^{*}\right)+2\sum_{\nu }R\left(\frac{\partial^{2}f(q_{0})}{\partial q^{\nu *}\partial q^{*}}\lambda^{\nu *}\lambda^{*}\right)+O(|\lambda {|}^{3})\;\mathrm{as}\;\lambda \to 0, \end{array}$$

*where ν*∈{1,*i*,*j*,*k*}, *and* ∂^2^*f*/∂*q*^*ν*^∂*q and* ∂^2^*f*/∂*q*^ν*^∂q* *are the second-order left GHR derivatives*.

**Proof.**

Using the result for ∂R(q)/∂q in table 1 and the chain rule in theorem 4.16, this corollary is proved similarly to the proof of corollary 4.21. ▪

**Theorem 4.25 (Taylor's theorem of centre kind)**

*Consider a third-order continuous real-differentiable function* f:S⊆H→R*. If q*_0_*,q*_0_+λ∈*S such that the segment joining them also lies in S, then*

4.48 $$\begin{array}{rl} f(q_{0}+\lambda )-f(q_{0}) & =\sum_{\mu }\frac{\partial f(q_{0})}{\partial q^{\mu }}\lambda^{\mu }+\frac{1}{2}\sum_{\mu ,\nu }\lambda^{\mu *}\frac{\partial^{2}f(q_{0})}{\partial q^{\nu }\partial q^{\mu *}}\lambda^{\nu }+O(\lambda^{3})\\ & =\sum_{\mu }\frac{\partial f(q_{0})}{\partial q^{\mu *}}\lambda^{\mu *}+\frac{1}{2}\sum_{\mu ,\nu }\lambda^{\mu }\frac{\partial^{2}f(q_{0})}{\partial q^{\nu *}\partial q^{\mu }}\lambda^{\nu *}+O(\lambda^{3})\;\mathrm{as}\;\lambda \to 0, \end{array}$$

*where μ,ν*∈{1,*i,j,k*}, *and* ∂^2^*f*/∂*q*^*ν*^∂*q*^μ*^ *and* ∂^2^*f*/∂*q*^*ν*^∂*q*^μ*^ *are the second-order left GHR derivatives.*

**Proof.**

Define an auxiliary function *g*(*t*)=*f*(*q*_0_+*t*λ) with 0≤*t*≤1. Using the chain rule in theorem 4.19 and using the property in (4.13), we obtain

4.49 $$g^{\prime }(t)=\sum_{\mu }\lambda^{\mu *}\frac{\partial_{r}\;f(q_{0}+t\lambda )}{\partial q^{\mu *}}=\sum_{\mu }\lambda^{\mu *}\frac{\partial f(q_{0}+t\lambda )}{\partial q^{\mu *}}.$$

Using the constant rule (4.3), we arrive at

4.50 $$g^{\prime \prime }(t)=\sum_{\mu }\lambda^{\mu *}{\left(\frac{\partial f(q_{0}+t\lambda )}{\partial q^{\mu *}}\right)}^{\prime }=\sum_{\mu ,\nu }\lambda^{\mu *}\frac{\partial^{2}f(q_{0}+t\lambda )}{\partial q^{\nu }\partial q^{\mu *}}\lambda^{\nu },$$

where *μ*,*ν*∈{1,*i*,*j*,*k*}. The rest of the proof is almost the same as that in theorem 4.23 and is thus omitted. ▪

**Remark 4.26**

The Taylor expansion in theorem 4.23 is concisely expressed using the GHR derivatives. This is different from the Taylor expansion given by Schwartz [18], which decomposes a quaternion *q* into two mutually perpendicular quaternions in a local coordinate system. In contrast, our approach treats the quaternion *q* as an augmented quaternion based on quaternion involutions [21]. Schwartz has also stated that his Taylor expansion would cause trouble when the function has terms *qωq*, where *ω* is a general quaternion. Note that there are no such restrictions in theorem 4.23, which only requires the real-differentiability condition in definition 3.1, that is, the functions *f*(*q*) should admit the partial derivatives with respect to the four real components *q*_*a*_,*q*_*b*_,*q*_*c*_ and *q*_*d*_.

## 5. Applications of the generalized HR calculus

We now illustrate the utility of the GHR calculus in optimization, statistics, signal processing, machine learning and other application areas.

### 5.1 Derivation of the widely linear quaternion least mean square algorithm

The widely linear QLMS (WL-QLMS) algorithm is based on the quaternion widely linear model *y*(*n*)=**w**^T^(*n*)**p**(*n*) which deals with the generality of quaternion signals (both proper and improper) [8,22,35], where **p**=(**x**^T^(*n*),**x**^*iT*^(*n*),**x**^*jT*^(*n*),**x**^*kT*^(*n*))^T^ is the augmented input vector and **w**=(**h**^T^(*n*),**g**^T^(*n*),**u**^T^(*n*),**v**^T^(*n*))^T^ is the associated weight (parameter) vector. The cost function to be minimized is a real-valued function of quaternion variables

5.1 $$J(n)=|e(n){|}^{2},$$

where *e*(*n*)=*d*(*n*)−*y*(*n*) is the error between the desired signal *d*(*n*) and the filter output *y*(*n*). The weight update of WL-QLMS is then given by

5.2 $$\Delta \text{w}(n)=\text{w}(n+1)-\text{w}(n)=-\alpha \nabla_{{\text{w}}^{*}}J(n)=-\alpha {\left(\frac{\partial J(n)}{\partial {\text{w}}^{*}}\right)}^{T},$$

where *α* is the step size, (⋅)^T^ denotes the transpose and the gradient $\nabla_{{\text{w}}^{*}}J(n)$ defines the direction of the maximum rate of change of *J* [13,36]. By the product rule within the GHR calculus, given in theorem 4.10, we have

5.3 $$\frac{\partial J}{\partial {\text{w}}^{*}}=e^{*}\frac{\partial e}{\partial {\text{w}}^{*}}+\frac{\partial e^{*}}{\partial {\text{w}}^{e*}}e,$$

where the time index ‘*n*’ is omitted. Note the convention that ∂*f*/∂**w** is a row vector whose *n*th element is ∂*f*/∂*w*_*n*_. The above GHR derivatives are calculated as

5.4 $$\begin{array}{rl} \frac{\partial e}{\partial {\text{w}}^{*}} & =\frac{\partial (d-{\text{w}}^{T}\text{p})}{\partial {\text{w}}^{*}}=-\frac{\partial ({\text{w}}^{T}\text{p})}{\partial {\text{w}}^{*}}=\frac{1}{2}{\text{p}}^{H}\\ \;\text{and}\;\;\frac{\partial e^{*}}{\partial {\text{w}}^{e*}}e & =\frac{\partial \left(d^{*}-{\text{p}}^{H}{\text{w}}^{*}\right)}{\partial {\text{w}}^{e*}}e=-\frac{\partial \left({\text{p}}^{H}{\text{w}}^{*}\right)}{\partial {\text{w}}^{e*}}e=-{\text{p}}^{H}R(e), \end{array}\}$$

where the terms ∂(*qν*)/∂q* and ∂(ωq*)/∂*q*^μ*^ are given in table 1 and are used in the last equalities in the expressions above. Substituting (5.4) into (5.3) yields

5.5 $$\frac{\partial J}{\partial {\text{w}}^{*}}=\frac{1}{2}e^{*}{\text{p}}^{H}-{\text{p}}^{H}R(e)=\left(\frac{1}{2}e^{*}-R(e)\right){\text{p}}^{H}=-\frac{1}{2}e{\text{p}}^{H}.$$

Finally, the update of the adaptive weight vector of WL-QLMS becomes

5.6 $$\text{w}(n+1)=\text{w}(n)+\alpha e(n){\text{p}}^{*}(n),$$

where the constant $\frac{1}{2}$ in (5.5) is absorbed into *α*.

**Remark 5.1**

There are many variations of WL-QLMS algorithms, such as the WL-QLMS algorithms based on variants {x*,**x**^i*^,**x**^j*^,**x**^k*^}, {**x**^*μ*^,**x**^*μi*^,**x**^*μj*^,**x**^*μk*^} and {x*,**x**^μi*^,**x**^μj*^,**x**^μk*^}. Note that if we start from *y*(*n*)=**w**^*H*^(*n*)**p**(*n*), the final update rule would become **w**(*n*+1)=**w**(*n*)+*α***p**(*n*)e*(*n*).

### 5.2 Derivation of quaternion nonlinear adaptive filtering algorithms

Tools of the GHR calculus allow us to concisely derive quaternion nonlinear adaptive filtering algorithms, a basis for fast-growing area of quaternion learning system. The same real-valued quadratic cost function as in real LMS and complex LMS is used, that is

5.7 $$J(n)=|e(n){|}^{2}=e_{a}^{2}(n)+e_{b}^{2}(n)+e_{c}^{2}(n)+e_{d}^{2}(n),$$

where *e*(*n*)=*e*_*a*_(*n*)+*ie*_*b*_(*n*)+*je*_*c*_(*n*)+*ke*_*d*_(*n*), *e*(*n*)=*d*(*n*)−*Φ*(*s*(*n*)), *s*(*n*)=**w**^T^(*n*)**x**(*n*), and *Φ* is the quaternion nonlinearity. The weight update is given by

5.8 $$\Delta \text{w}(n)=\text{w}(n+1)-\text{w}(n)=-\alpha \nabla_{{\text{w}}^{*}}J(n)=-\alpha {\left(\frac{\partial J(n)}{\partial {\text{w}}^{*}}\right)}^{T},$$

where *α*>0 is the real step size, (⋅)^T^ denotes the transpose, and the gradient $\nabla_{{\text{w}}^{*}}J(n)$ defines the direction of the maximum rate of change of *J* [13,36]. By using the chain rule in theorem 4.16, the above gradient can be calculated as

5.9 $$\frac{\partial J}{\partial {\text{w}}^{*}}=\sum_{\mu \in \{1,i,j,k\}}\frac{\partial J}{\partial s^{\mu *}}\frac{\partial s^{\mu *}}{\partial {\text{w}}^{*}},$$

where time index ‘*n*’ is omitted for convenience. Note the convention that ∂*f*/∂**w** is a row vector whose *n*th element is ∂*f*/∂*w*_*n*_. Using the term ∂(ωq*)/∂q* in table 1, we have

5.10 $$\frac{\partial s^{*}}{\partial {\text{w}}^{*}}=\frac{\partial ({\text{x}}^{H}{\text{w}}^{*})}{\partial {\text{w}}^{*}}={\text{x}}^{H}.$$

Upon applying the second equality in (4.12) and using the term ∂(ωq*)/∂*q*^μ*^μ in table 1, this yields

5.11 $$\frac{\partial s^{\mu *}}{\partial {\text{w}}^{*}}={\left(\frac{\partial s^{*}}{\partial {\text{w}}^{\mu *}}\right)}^{\mu }=-\mu \frac{\partial \left({\text{x}}^{H}{\text{w}}^{*}\right)}{\partial {\text{w}}^{\mu *}}\mu =-\mu {\text{x}}^{H}R(\mu )=0,\;\forall \;\mu \in \{i,j,k\}.$$

Using the chain rule in corollary 4.18, we have

5.12 $$\begin{array}{rl} \frac{\partial J}{\partial s^{*}} & =2e_{a}\frac{\partial e_{a}}{\partial s^{*}}+2e_{b}\frac{\partial e_{b}}{\partial s^{*}}+2e_{c}\frac{\partial e_{c}}{\partial s^{*}}+2e_{d}\frac{\partial e_{d}}{\partial s^{*}}\\ & =-2e_{a}\frac{\partial \Phi_{a}(s)}{\partial s^{*}}-2e_{b}\frac{\partial \Phi_{b}(s)}{\partial s^{*}}-2e_{c}\frac{\partial \Phi_{c}(s)}{\partial s^{*}}-2e_{d}\frac{\partial \Phi_{d}(s)}{\partial s^{*}}. \end{array}$$

Upon substituting (5.10)–(5.12) into (5.9), we arrive at

5.13 $$\frac{\partial J}{\partial {\text{w}}^{*}}=-2\left(e_{a}\frac{\partial \Phi_{a}(s)}{\partial s^{*}}+e_{b}\frac{\partial \Phi_{b}(s)}{\partial s^{*}}+e_{c}\frac{\partial \Phi_{c}(s)}{\partial s^{*}}+e_{d}\frac{\partial \Phi_{d}(s)}{\partial s^{*}}\right){\text{x}}^{H}.$$

Finally, the weight update for this quaternion nonlinear adaptive filtering algorithm becomes

5.14 $$\Delta \text{w}(n)=\alpha \left(e_{a}(n)\frac{\partial \Phi_{a}(s(n))}{\partial s^{*}(n)}+e_{b}(n)\frac{\partial \Phi_{b}(s(n))}{\partial s^{*}(n)}+e_{c}(n)\frac{\partial \Phi_{c}(s(n))}{\partial s^{*}(n)}+e_{d}(n)\frac{\partial \Phi_{d}(s(n))}{\partial s^{*}(n)}\right){\text{x}}^{*}(n),$$

where the constant 2 in (5.13) is absorbed into the step size *α*.

For illustration, consider an example where *Φ* is a nonlinear split-quaternion function *Φ*(*s*)=*φ*(*s*_*a*_)+*iφ*(*s*_*b*_)+*jφ*(*s*_*c*_)+*φ*(*s*_*d*_) and φ:R→R is a real-valued differentiable function. Then,

5.15 $$\frac{\partial \Phi_{a}(s)}{\partial s^{*}}=\frac{\partial \varphi (s_{a})}{\partial s^{*}}=\frac{1}{4}\varphi^{\prime }(s_{a}).$$

In a similar manner, we have

5.16 $$\frac{\partial \Phi_{b}(s)}{\partial s^{*}}=\frac{i}{4}\varphi^{\prime }(s_{b}),\;\frac{\partial \Phi_{c}(s)}{\partial s^{*}}=\frac{j}{4}\varphi^{\prime }(s_{c})\;\mathrm{and}\;\frac{\partial \Phi_{d}(s)}{\partial s^{*}}=\frac{k}{4}\varphi^{\prime }(s_{d}).$$

The weight update for such a quaternion nonlinear adaptive filtering algorithm becomes

5.17 $$\Delta \text{w}(n)=\alpha (e_{a}(n)\varphi^{\prime }(s_{a}(n))+ie_{b}(n)\varphi^{\prime }(s_{b}(n))+je_{c}(n)\varphi^{\prime }(s_{c}(n))+ke_{d}(n)\varphi^{\prime }(s_{d}(n))){\text{x}}^{*}(n),$$

where the constant $\frac{1}{4}$ in (5.15) and (5.16) is absorbed into the step size *α*.

Another example is when *Φ* is a quaternion linear function *Φ*(*s*)=*s*, that is *φ*(*x*)=*x* in (5.17). Then, the update of the adaptive weight vector within the QLMS algorithm becomes

5.18 $$\text{w}(n+1)=\text{w}(n)+\alpha e(n){\text{x}}^{*}(n)$$

illustrating the generic nature of the GHR calculus.

**Remark 5.2**

From (5.17) and (5.18), we note that quaternion linear and nonlinear adaptive filtering algorithms have been developed in a unified form. This also shows that the GHR calculus gives us much more freedom in the design, as the nonlinear function *Φ* is not required to satisfy the odd-symmetry condition [28,37]. We can derive many other algorithms, such as the augmented quaternion nonlinear gradient descent algorithm [38], in the same way. In the interest of space, we leave this to the interested reader.

**Remark 5.3**

The QLMS algorithm (5.18) is different from the QLMS in [4,13], due to the use of different product rule. The traditional product rule was used in [4,13] to simplify the derivation; however, our examples in §3a illustrate that the traditional product rule is not applicable to the HR derivatives. On the other hand, the chain rules within the GHR calculus result in the QLMS in (5.18), which has the same generic form as that of the complex LMS [39]. For the performance comparison and steady-state analysis of the existing QLMS algorithms, we refer the reader to [40] for more details.

## 6. Conclusion

A novel and rigorous framework for the efficient computation of quaternion derivatives, referred to as the GHR calculus, has been established. The GHR methodology has been shown to greatly relax the existence conditions for the derivatives of general nonlinear functions of quaternion variables, and to simplify the calculation of quaternion derivatives through its novel product and chain rules. We have shown that, unlike the existing quaternion derivatives, the GHR calculus is general and can be used for both analytic and non-analytic functions of quaternion variables. The core of the GHR calculus is the use of quaternion rotations in order to overcome the non-commutativity of quaternion product, and the use of quaternion involutions to obtain an elegant quaternion basis. Through the analysis and examples, the proposed framework has been shown to allow for real- and complex-valued optimization algorithms to be extended to the quaternion field in a generic, compact and intuitive way. Application case studies in statistical signal processing and learning systems demonstrate the effectiveness of the proposed GHR framework.

## Appendices

Several results and proofs of statements from the main body are next detailed.

## Appendix A. The derivation of the HR calculus

The following lemmas are useful to distinguish between the GHR derivatives and the standard quaternion differential.

Lemma A.1 *Let* $f_{n}:H\to H\;(n=1,2,3,4)$ *be any arbitrary quaternion-valued functions and* μ∈H,μ≠0. *If for the left case*

A 1 $$f_{1}dq^{\mu }+f_{2}dq^{\mu i}+f_{3}dq^{\mu j}+f_{4}dq^{\mu k}=0$$

*or for the right case*

A 2 $$dq^{\mu }f_{1}+dq^{\mu i}f_{2}+dq^{\mu j}f_{3}+dq^{\mu k}f_{4}=0,$$

*then f*_*n*_=0 *for n*∈{1,2,3,4}.

Proof. The left case. By applying the rotation transformation on both sides of (2.6), it follows that

A 3 $$\begin{array}{rl} q^{\mu } & =q_{a}+i^{\mu }q_{b}+j^{\mu }q_{c}+k^{\mu }q_{d},\;q^{\mu i}=q_{a}+i^{\mu }q_{b}-j^{\mu }q_{c}-k^{\mu }q_{d}\\ q^{\mu j} & =q_{a}-i^{\mu }q_{b}+j^{\mu }q_{c}-k^{\mu }q_{d},\;q^{\mu k}=q_{a}-i^{\mu }q_{b}-j^{\mu }q_{c}+k^{\mu }q_{d}. \end{array}\}$$

Upon applying the differential operator to the above expressions and substituting *dq*^*μ*^,*dq*^*μi*^,*dq*^*μj*^ and *dq*^*μk*^ into (A 1), we have

A 4 $$\begin{array}{rl} & f_{1}(dq_{a}+i^{\mu }dq_{b}+j^{\mu }dq_{c}+k^{\mu }dq_{d})+f_{2}(dq_{a}+i^{\mu }dq_{b}-j^{\mu }dq_{c}-k^{\mu }dq_{d})\\ & \;+f_{3}(dq_{a}-i^{\mu }dq_{b}+j^{\mu }dq_{c}-k^{\mu }dq_{d})+f_{4}(dq_{a}-i^{\mu }dq_{b}-j^{\mu }dq_{c}+k^{\mu }dq_{d})=0. \end{array}$$

This is equivalent to

A 5 $$\begin{array}{rl} & (\;f_{1}+f_{2}+f_{3}+f_{4})dq_{a}+(\;f_{1}+f_{2}-f_{3}-f_{4})i^{\mu }dq_{b}\\ & \;+(\;f_{1}-f_{2}+f_{3}-f_{4})j^{\mu }dq_{c}+(\;f_{1}-f_{2}-f_{3}+f_{4})k^{\mu }dq_{d}=0. \end{array}$$

Since the differentials *dq*_*a*_,*dq*_*b*_,*dq*_*c*_ and *dq*_*d*_ are independent, this yields

A 6 $$\begin{array}{rl} f_{1}+f_{2}+f_{3}+f_{4} & =0,\;f_{1}+f_{2}-f_{3}-f_{4}=0,\\ \;f_{1}-f_{2}+f_{3}-f_{4} & =0,\;f_{1}-f_{2}-f_{3}+f_{4}=0. \end{array}\}$$

Hence, it follows that *f*_1_=*f*_2_=*f*_3_=*f*_4_=0. The right case can be proved in a similar way. ▪

Lemma A.2 *Let* $f_{n}:H\to H\;(n=1,2,3,4)$ *be any arbitrary quaternion-valued function and* μ∈H,μ≠0. *If for the left case*

$$f_{1}dq^{\mu *}+f_{2}dq^{\mu i*}+f_{3}dq^{\mu j*}+f_{4}dq^{\mu k*}=0$$

*or for the right case*

$$dq^{\mu *}f_{1}+dq^{\mu i*}f_{2}+dq^{\mu j*}f_{3}+dq^{\mu k*}f_{4}=0,$$

*then f*_*n*_=0 *for n*∈{1,2,3,4}.

Proof. The proof of lemma A.2 follows from that of lemma A.1 and is omitted. ▪

We now provide the derivation of the HR calculus. For any quaternion-valued function f(q)∈H, we can state (since the fields H and $R^{4}$ are isomorphic) that

A 7 $$f(q)=f_{a}(q_{a},q_{b},q_{c},q_{d})+if_{b}(q_{a},q_{b},q_{c},q_{d})+jf_{c}(q_{a},q_{b},q_{c},q_{d})+kf_{d}(q_{a},q_{b},q_{c},q_{d}),$$

where $f_{a}(\cdot ),\;f_{b}(\cdot ),\;f_{c}(\cdot ),\;f_{d}(\cdot )\in R$. Then, the function *f* can be equally seen as a function of the four independent real variables *q*_*a*_,*q*_*b*_,*q*_*c*_ and *q*_*d*_, and the differential of *f* can be expressed as [10]

A 8 $$[\mathrm{Left}]:\;df=\frac{\partial f}{\partial q_{a}}dq_{a}+\frac{\partial f}{\partial q_{b}}dq_{b}+\frac{\partial f}{\partial q_{c}}dq_{c}+\frac{\partial f}{\partial q_{d}}dq_{d}$$

and

A 9 $$[\mathrm{Right}]:\;df=dq_{a}\frac{\partial f}{\partial q_{a}}+dq_{b}\frac{\partial f}{\partial q_{b}}+dq_{c}\frac{\partial f}{\partial q_{c}}+dq_{d}\frac{\partial f}{\partial q_{d}},$$

where ∂*f*/∂*q*_*a*_, ∂*f*/∂*q*_*b*_, ∂*f*/∂*q*_*c*_ and ∂*f*/∂*q*_*d*_ are the partial derivatives of *f* with respect to *q*_*a*_, *q*_*b*_, *q*_*c*_ and *q*_*d*_, respectively. Note that the two equations are identical since *dq*_*a*_, *dq*_*b*_, *dq*_*c*_ and *dq*_*d*_ are real quantities. As a result, both equations are equally valid as a starting point for the derivation of the HR calculus.

*The left case* (A 8). There are two ways to link the real and quaternion differentials; these are based on (2.7) and its conjugate which correspond to the HR derivatives and conjugate HR derivatives.

**A. 1. The left HR derivatives**

From (2.7), the differentials of the components of a quaternion can be formulated as

A 10 $$\begin{array}{rl} dq_{a} & =\frac{1}{4}(dq+dq^{i}+dq^{j}+dq^{k}),\;dq_{b}=-\frac{i}{4}(dq+dq^{i}-dq^{j}-dq^{k})\\ dq_{c} & =-\frac{j}{4}(dq-dq^{i}+dq^{j}-dq^{k}),\;dq_{d}=-\frac{k}{4}(dq-dq^{i}-dq^{j}+dq^{k}). \end{array}\}$$

By inserting (A 10) into (A 8), the differential of the function *f* becomes

A 11 $$\begin{array}{rl} df & =\frac{1}{4}\frac{\partial f}{\partial q_{a}}(dq+dq^{i}+dq^{j}+dq^{k})-\frac{1}{4}\frac{\partial f}{\partial q_{b}}i(dq+dq^{i}-dq^{j}-dq^{k})\\ & \;-\frac{1}{4}\frac{\partial f}{\partial q_{c}}j(dq-dq^{i}+dq^{j}-dq^{k})-\frac{1}{4}\frac{\partial f}{\partial q_{d}}k(dq-dq^{i}-dq^{j}+dq^{k})\\ & =\frac{1}{4}\left(\frac{\partial f}{\partial q_{a}}-\frac{\partial f}{\partial q_{b}}i-\frac{\partial f}{\partial q_{c}}j-\frac{\partial f}{\partial q_{d}}k\right)dq+\frac{1}{4}\left(\frac{\partial f}{\partial q_{a}}-\frac{\partial f}{\partial q_{b}}i+\frac{\partial f}{\partial q_{c}}j+\frac{\partial f}{\partial q_{d}}k\right)dq^{i}\\ & \;+\frac{1}{4}\left(\frac{\partial f}{\partial q_{a}}+\frac{\partial f}{\partial q_{b}}i-\frac{\partial f}{\partial q_{c}}j+\frac{\partial f}{\partial q_{d}}k\right)dq^{j}+\frac{1}{4}\left(\frac{\partial f}{\partial q_{a}}+\frac{\partial f}{\partial q_{b}}i+\frac{\partial f}{\partial q_{c}}j-\frac{\partial f}{\partial q_{d}}k\right)dq^{k}. \end{array}$$

Now, we can define the formal left derivatives ∂*f*/∂*q*,∂*f*/∂*q*^*i*^,∂*f*/∂*q*^*j*^ and ∂*f*/∂*q*^*k*^, so that

A 12 $$df=\frac{\partial f}{\partial q}dq+\frac{\partial f}{\partial q^{i}}dq^{i}+\frac{\partial f}{\partial q^{j}}dq^{j}+\frac{\partial f}{\partial q^{k}}dq^{k}$$

holds. Comparing (A 12) with (A 11) and applying lemma A.1, yields the left HR derivatives

A 13 $${\left[\begin{array}{c} \frac{\partial f}{\partial q}\\ \frac{\partial f}{\partial q^{i}}\\ \frac{\partial f}{\partial q^{j}}\\ \frac{\partial f}{\partial q^{k}} \end{array}\right]}^{T}=\frac{1}{4}{\left[\begin{array}{c} \frac{\partial f}{\partial q_{a}}\\ \frac{\partial f}{\partial q_{b}}\\ \frac{\partial f}{\partial q_{c}}\\ \frac{\partial f}{\partial q_{d}} \end{array}\right]}^{T}\left[\begin{array}{cccc} 1 & 1 & 1 & 1\\ -i & -i & i & i\\ -j & j & -j & j\\ -k & k & k & -k \end{array}\right].$$

**A. 2. The left conjugate HR derivatives**

Upon applying the conjugate operator to both sides of (2.7), the differentials of the components of a quaternion can be formulated as

A 14 $$\begin{array}{rl} dq_{a} & =\frac{1}{4}(dq^{*}+dq^{i*}+dq^{j*}+dq^{k*}),\;dq_{b}=\frac{i}{4}(dq^{*}+dq^{i*}-dq^{j*}-dq^{k*}),\\ \;dq_{c} & =\frac{j}{4}(dq^{*}-dq^{i*}+dq^{j*}-dq^{k*}),\;dq_{d}=\frac{k}{4}(dq^{*}-dq^{i*}-dq^{j*}+dq^{k*}). \end{array}\}$$

By inserting (A 14) into (A 8), the differential of *f* can be written as

A 15 $$\begin{array}{rl} df & =\frac{1}{4}\frac{\partial f}{\partial q_{a}}(dq^{*}+dq^{i*}+dq^{j*}+dq^{k*})+\frac{1}{4}\frac{\partial f}{\partial q_{b}}i(dq^{*}+dq^{i*}-dq^{j*}-dq^{k*})\\ & \;+\frac{1}{4}\frac{\partial f}{\partial q_{c}}j(dq^{*}-dq^{i*}+dq^{j*}-dq^{k*})+\frac{1}{4}\frac{\partial f}{\partial q_{d}}k(dq^{*}-dq^{i*}-dq^{j*}+dq^{k*})\\ & =\frac{1}{4}\left(\frac{\partial f}{\partial q_{a}}+\frac{\partial f}{\partial q_{b}}i+\frac{\partial f}{\partial q_{c}}j+\frac{\partial f}{\partial q_{d}}k\right)dq^{*}+\frac{1}{4}\left(\frac{\partial f}{\partial q_{a}}+\frac{\partial f}{\partial q_{b}}i-\frac{\partial f}{\partial q_{c}}j-\frac{\partial f}{\partial q_{d}}k\right)dq^{i*}\\ & \;+\frac{1}{4}\left(\frac{\partial f}{\partial q_{a}}-\frac{\partial f}{\partial q_{b}}i+\frac{\partial f}{\partial q_{c}}j-\frac{\partial f}{\partial q_{d}}k\right)dq^{j*}+\frac{1}{4}\left(\frac{\partial f}{\partial q_{a}}-\frac{\partial f}{\partial q_{b}}i-\frac{\partial f}{\partial q_{c}}j+\frac{\partial f}{\partial q_{d}}k\right)dq^{k*}. \end{array}$$

We can now define the formal left derivatives ∂*f*/∂*q**,∂*f*/∂*q*^*i**^,∂*f*/∂*q*^*j**^ and ∂*f*/∂*q*^*k**^, so that

A 16 $$df=\frac{\partial f}{\partial q^{*}}dq^{*}+\frac{\partial f}{\partial q^{i*}}dq^{i*}+\frac{\partial f}{\partial q^{j*}}dq^{j*}+\frac{\partial f}{\partial q^{k}}dq^{k*}$$

holds. Upon comparing (A 16) with (A 15) and applying lemma A.2, the following left conjugate HR derivatives are obtained:

A 17 $${\left[\begin{array}{c} \frac{\partial f}{\partial q^{*}}\\ \frac{\partial f}{\partial q^{i*}}\\ \frac{\partial f}{\partial q^{j*}}\\ \frac{\partial f}{\partial q^{k*}} \end{array}\right]}^{T}=\frac{1}{4}{\left[\begin{array}{c} \frac{\partial f}{\partial q_{a}}\\ \frac{\partial f}{\partial q_{b}}\\ \frac{\partial f}{\partial q_{c}}\\ \frac{\partial f}{\partial q_{d}} \end{array}\right]}^{T}\left[\begin{array}{cccc} 1 & 1 & 1 & 1\\ i & i & -i & -i\\ j & -j & j & -j\\ k & -k & -k & k \end{array}\right].$$

*The right case* (A 9). There are two ways to link the real and quaternion differentials, the approach in (2.7) and its conjugate, which induce to the right HR derivatives or conjugate right HR derivatives, respectively.

**A. 3. The right HR derivatives**

Applying a rotation transformation to both sides of (A 10), we have

A 18 $$\begin{array}{rl} dq_{a} & =\frac{1}{4}(dq+dq^{i}+dq^{j}+dq^{k}),\;dq_{b}=-\frac{1}{4}(dq+dq^{i}-dq^{j}-dq^{k})i,\\ dq_{c} & =-\frac{1}{4}(dq-dq^{i}+dq^{j}-dq^{k})j,\;dq_{d}=-\frac{1}{4}(dq-dq^{i}-dq^{j}+dq^{k})k. \end{array}\}$$

Then, by substituting (A 18) into (A 9), the differential of *f* becomes

A 19 $$\begin{array}{rl} df & =\frac{1}{4}(dq+dq^{i}+dq^{j}+dq^{k})\frac{\partial f}{\partial q_{a}}-\frac{1}{4}(dq+dq^{i}-dq^{j}-dq^{k})i\frac{\partial f}{\partial q_{b}}\\ & \;-\frac{1}{4}(dq-dq^{i}+dq^{j}-dq^{k})j\frac{\partial f}{\partial q_{c}}-\frac{1}{4}(dq-dq^{i}-dq^{j}+dq^{k})k\frac{\partial f}{\partial q_{d}}\\ & =\frac{1}{4}dq\left(\frac{\partial f}{\partial q_{a}}-i\frac{\partial f}{\partial q_{b}}-j\frac{\partial f}{\partial q_{c}}-k\frac{\partial f}{\partial q_{d}}\right)+\frac{1}{4}dq^{i}\left(\frac{\partial f}{\partial q_{a}}-i\frac{\partial f}{\partial q_{b}}+j\frac{\partial f}{\partial q_{c}}+k\frac{\partial f}{\partial q_{d}}\right)\\ & \;+\frac{1}{4}dq^{j}\left(\frac{\partial f}{\partial q_{a}}+i\frac{\partial f}{\partial q_{b}}-j\frac{\partial f}{\partial q_{c}}+k\frac{\partial f}{\partial q_{d}}\right)+\frac{1}{4}dq^{k}\left(\frac{\partial f}{\partial q_{a}}+i\frac{\partial f}{\partial q_{b}}+j\frac{\partial f}{\partial q_{c}}-k\frac{\partial f}{\partial q_{d}}\right). \end{array}$$

Now, define the formal right derivatives ∂_*r*_*f*/∂*q*,∂_*r*_*f*/∂*q*^*i*^,∂_*r*_*f*/∂*q*^*j*^ and ∂_*r*_*f*/∂*q*^*k*^, so that

A 20 $$df=dq\frac{\partial_{r}\;f}{\partial q}+dq^{i}\frac{\partial_{r}\;f}{\partial q^{i}}+dq^{j}\frac{\partial_{r}\;f}{\partial q^{j}}+dq^{k}\frac{\partial_{r}\;f}{\partial q^{k}}$$

holds. Comparing (A 20) and (A 19) and using lemma A.1, the following right HR derivatives are obtained:

A 21 $$\left[\begin{array}{c} \frac{\partial_{r}\;f}{\partial q}\\ \frac{\partial_{r}\;f}{\partial q^{i}}\\ \frac{\partial_{r}\;f}{\partial q^{j}}\\ \frac{\partial_{r}\;f}{\partial q^{k}} \end{array}\right]=\frac{1}{4}\left[\begin{array}{cccc} 1 & -i & -j & -k\\ 1 & -i & j & k\\ 1 & i & -j & k\\ 1 & i & j & -k \end{array}\right]\;\left[\begin{array}{c} \frac{\partial f}{\partial q_{a}}\\ \frac{\partial f}{\partial q_{b}}\\ \frac{\partial f}{\partial q_{c}}\\ \frac{\partial f}{\partial q_{d}} \end{array}\right].$$

**A. 4. The right conjugate HR derivatives**

By applying a rotation transformation to both sides of (A 14), we have

A 22 $$\begin{array}{rl} dq_{a} & =\frac{1}{4}(dq^{*}+dq^{i*}+dq^{j*}+dq^{k*}),\;dq_{b}=\frac{1}{4}(dq^{*}+dq^{i*}-dq^{j*}-dq^{k*})i,\\ dq_{c} & =\frac{1}{4}(dq^{*}-dq^{i*}+dq^{j*}-dq^{k*})j,\;dq_{d}=\frac{1}{4}(dq^{*}-dq^{i*}-dq^{j*}+dq^{k*})k. \end{array}\}$$

Upon substituting (A 22) into (A 9), the differential of *f* can be written as

A 23 $$\begin{array}{rl} df & =\frac{1}{4}(dq^{*}+dq^{i*}+dq^{j*}+dq^{k*})\frac{\partial f}{\partial q_{a}}+\frac{1}{4}(dq^{*}+dq^{i*}-dq^{j*}-dq^{k*})i\frac{\partial f}{\partial q_{b}}\\ & \;+\frac{1}{4}(dq^{*}-dq^{i*}+dq^{j*}-dq^{k*})j\frac{\partial f}{\partial q_{c}}+\frac{1}{4}(dq^{*}-dq^{i*}-dq^{j*}+dq^{k*})k\frac{\partial f}{\partial q_{d}}\\ & =\frac{1}{4}dq^{*}\left(\frac{\partial f}{\partial q_{a}}+i\frac{\partial f}{\partial q_{b}}+j\frac{\partial f}{\partial q_{c}}+k\frac{\partial f}{\partial q_{d}}\right)+\frac{1}{4}dq^{i*}\left(\frac{\partial f}{\partial q_{a}}+i\frac{\partial f}{\partial q_{b}}-j\frac{\partial f}{\partial q_{c}}-k\frac{\partial f}{\partial q_{d}}\right)\\ & \;+\frac{1}{4}dq^{j*}\left(\frac{\partial f}{\partial q_{a}}-i\frac{\partial f}{\partial q_{b}}+j\frac{\partial f}{\partial q_{c}}-k\frac{\partial f}{\partial q_{d}}\right)+\frac{1}{4}dq^{k*}\left(\frac{\partial f}{\partial q_{a}}-i\frac{\partial f}{\partial q_{b}}-j\frac{\partial f}{\partial q_{c}}+k\frac{\partial f}{\partial q_{d}}\right). \end{array}$$

Now, define the formal right derivatives ∂_*r*_*f*/∂*q**,∂_*r*_*f*/∂*q*^*i**^,∂_*r*_*f*/∂*q*^*j**^ and ∂_*r*_*f*/∂*q*^*k**^, so that

A 24 $$df=dq^{*}\frac{\partial_{r}\;f}{\partial q^{*}}+dq^{i*}\frac{\partial_{r}\;f}{\partial q^{i*}}+dq^{j*}\frac{\partial_{r}\;f}{\partial q^{j*}}+dq^{k*}\frac{\partial_{r}\;f}{\partial q^{k*}}$$

holds. Comparing (A 24) with (A 23) and using lemma A.2, we obtain the following right conjugate HR derivatives:

A 25 $$\left[\begin{array}{c} \frac{\partial_{r}\;f}{\partial q^{*}}\\ \frac{\partial_{r}\;f}{\partial q^{i*}}\\ \frac{\partial_{r}\;f}{\partial q^{j*}}\\ \frac{\partial_{r}\;f}{\partial q^{k*}} \end{array}\right]=\frac{1}{4}\left[\begin{array}{cccc} 1 & i & j & k\\ 1 & i & -j & -k\\ 1 & -i & j & -k\\ 1 & -i & -j & k \end{array}\right]\;\left[\begin{array}{c} \frac{\partial f}{\partial q_{a}}\\ \frac{\partial f}{\partial q_{b}}\\ \frac{\partial f}{\partial q_{c}}\\ \frac{\partial f}{\partial q_{d}} \end{array}\right].$$

## Appendix B. The proof of the product rule

Within the GHR calculus, when a quaternion function is post-multiplied by a real function, the novel product rule degenerates into the traditional product rule. This is stated in the next lemma.

Lemma B.1 *If the functions* f:H→H *and* g:H→R *have the left GHR derivatives, then their product fg satisfies the traditional product rule*

B 1 $$\frac{\partial (\;fg)}{\partial q^{\mu }}=f\frac{\partial g}{\partial q^{\mu }}+\frac{\partial f}{\partial q^{\mu }}g\;and\;\frac{\partial (\;fg)}{\partial q^{\mu *}}=f\frac{\partial g}{\partial q^{\mu *}}+\frac{\partial f}{\partial q^{\mu *}}g,$$

*where* ∂*f*/∂*q*^*μ*^ *and* ∂*f*/∂*q*^*μ**^ *are the left GHR derivatives in definition 4.1*.

Proof. Let *f*=*f*_*a*_+*if*_*b*_+*jf*_*c*_+*kf*_*d*_, where $f_{a},f_{b},f_{c},f_{d}\in R$, then *fg*=*f*_*a*_*g*+*if*_*b*_*g*+*jf*_*c*_*g*+*kf*_*d*_*g*. Using the property ∂(*νf*)/∂*q*^*μ*^=*ν*(∂*f*/∂*q*^*μ*^) in (4.3), we have

B 2 $$\begin{array}{rl} \frac{\partial (\;fg)}{\partial q^{\mu }} & =\frac{\partial ((\;f_{a}+if_{b}+jf_{c}+kf_{d})g)}{\partial q^{\mu }}\\ & =\frac{\partial (\;f_{a}g)}{\partial q^{\mu }}+i\frac{\partial (\;f_{b}g)}{\partial q^{\mu }}+j\frac{\partial (\;f_{c}g)}{\partial q^{\mu }}+k\frac{\partial (\;f_{d}g)}{\partial q^{\mu }}\\ & =f_{a}\frac{\partial g}{\partial q^{\mu }}+if_{b}\frac{\partial g}{\partial q^{\mu }}+jf_{c}\frac{\partial g}{\partial q^{\mu }}+kf_{d}\frac{\partial g}{\partial q^{\mu }}+\frac{\partial f_{a}}{\partial q^{\mu }}g+i\frac{\partial f_{b}}{\partial q^{\mu }}g+j\frac{\partial f_{c}}{\partial q^{\mu }}g+k\frac{\partial f_{d}}{\partial q^{\mu }}g\\ & =f\frac{\partial g}{\partial q^{\mu }}+\left(\frac{\partial f_{a}}{\partial q^{\mu }}+i\frac{\partial f_{b}}{\partial q^{\mu }}+j\frac{\partial f_{c}}{\partial q^{\mu }}+k\frac{\partial f_{d}}{\partial q^{\mu }}\right)g=f\frac{\partial g}{\partial q^{\mu }}+\frac{\partial f}{\partial q^{\mu }}g. \end{array}$$

Hence, the first part of the lemma is proved, and the rest follows in a similar way. ▪

The proof of theorem 4.10 From (2.5), note that {1,*i*^*μ*^,*j*^*μ*^,*k*^*μ*^} is another orthogonal basis of H. Then, the quaternion-valued function *g* can be expressed in the following way:

B 3 $$g=g_{a}+i^{\mu }g_{b}+j^{\mu }g_{c}+k^{\mu }g_{d},\;g_{a},g_{b},g_{c},g_{d}\in R,$$

and *fg*=*fg*_*a*_+*fi*^*μ*^*g*_*b*_+*fj*^*μ*^*g*_*c*_+*fk*^*μ*^*g*_*d*_, f∈H. Using the sum rule, it follows that

B 4 $$\frac{\partial (\;fg)}{\partial q^{\mu }}=\frac{\partial (\;fg_{a})}{\partial q^{\mu }}+\frac{\partial (\;fi^{\mu }g_{b})}{\partial q^{\mu }}+\frac{\partial (\;fi^{\mu }g_{c})}{\partial q^{\mu }}+\frac{\partial (\;fi^{\mu }g_{d})}{\partial q^{\mu }}.$$

Applying lemma B.1 to the right side of (B 4) yields

B 5 $$\begin{array}{rl} \frac{\partial (\;fg)}{\partial q^{\mu }} & =f\frac{\partial g_{a}}{\partial q^{\mu }}+fi^{\mu }\frac{\partial g_{b}}{\partial q^{\mu }}+fj^{\mu }\frac{\partial g_{c}}{\partial q^{\mu }}+fk^{\mu }\frac{\partial g_{d}}{\partial q^{\mu }}+\frac{\partial f}{\partial q^{\mu }}g_{a}+\frac{\partial (\;fi^{\mu })}{\partial q^{\mu }}g_{b}+\frac{\partial (\;fj^{\mu })}{\partial q^{\mu }}g_{c}+\frac{\partial (\;fk^{\mu })}{\partial q^{\mu }}g_{d}\\ & =f\left(\frac{\partial g_{a}}{\partial q^{\mu }}+i^{\mu }\frac{\partial g_{b}}{\partial q^{\mu }}+j^{\mu }\frac{\partial g_{c}}{\partial q^{\mu }}+k^{\mu }\frac{\partial g_{d}}{\partial q^{\mu }}\right)+\frac{\partial f}{\partial q^{\mu }}g_{a}+\frac{\partial (\;fi^{\mu })}{\partial q^{\mu }}g_{b}+\frac{\partial (\;fj^{\mu })}{\partial q^{\mu }}g_{c}+\frac{\partial (\;fk^{\mu })}{\partial q^{\mu }}g_{d}\\ & =f\frac{\partial g}{\partial q^{\mu }}+\frac{\partial f}{\partial q^{\mu }}g_{a}+\frac{\partial (\;fi^{\mu })}{\partial q^{\mu }}g_{b}+\frac{\partial (\;fj^{\mu })}{\partial q^{\mu }}g_{c}+\frac{\partial (\;fk^{\mu })}{\partial q^{\mu }}g_{d}. \end{array}$$

Next, by using the result ∂( *fν*)/∂*q*^*μ*^=(∂*f*/∂*q*^*νμ*^)*ν* in (4.3), we have

B 6 $$\begin{array}{rl} \frac{\partial (\;fg)}{\partial q^{\mu }} & =f\frac{\partial g}{\partial q^{\mu }}+\frac{\partial f}{\partial q^{\mu }}g_{a}+\frac{\partial f}{\partial q^{\mu i}}i^{\mu }g_{b}+\frac{\partial f}{\partial q^{\mu j}}j^{\mu }g_{c}+\frac{\partial f}{\partial q^{\mu k}}k^{\mu }g_{d}\\ & =f\frac{\partial g}{\partial q^{\mu }}+\frac{1}{4}\left(\frac{\partial f}{\partial q_{a}}-\frac{\partial f}{\partial q_{b}}i^{\mu }-\frac{\partial f}{\partial q_{c}}j^{\mu }-\frac{\partial f}{\partial q_{d}}k^{\mu }\right)g_{a}+\frac{1}{4}\left(\frac{\partial f}{\partial q_{a}}-\frac{\partial f}{\partial q_{b}}i^{\mu }+\frac{\partial f}{\partial q_{c}}j^{\mu }+\frac{\partial f}{\partial q_{d}}k^{\mu }\right)i^{\mu }g_{b}\\ & \;+\frac{1}{4}\left(\frac{\partial f}{\partial q_{a}}+\frac{\partial f}{\partial q_{b}}i^{\mu }-\frac{\partial f}{\partial q_{c}}j^{\mu }+\frac{\partial f}{\partial q_{d}}k^{\mu }\right)j^{\mu }g_{c}+\frac{1}{4}\left(\frac{\partial f}{\partial q_{a}}+\frac{\partial f}{\partial q_{b}}i^{\mu }+\frac{\partial f}{\partial q_{c}}j^{\mu }-\frac{\partial f}{\partial q_{d}}k^{\mu }\right)k^{\mu }g_{d}, \end{array}$$

where definition 4.1 and (2.4) were used in the last equality above. Grouping together ∂*f*/∂*q*_*a*_, ∂*f*/∂*q*_*b*_, ∂*f*/∂*q*_*c*_ and ∂*f*/∂*q*_*d*_ in (B 7) finally yields

B 7 $$\begin{array}{rl} \frac{\partial (\;fg)}{\partial q^{\mu }} & =f\frac{\partial g}{\partial q^{\mu }}+\frac{1}{4}\frac{\partial f}{\partial q_{a}}\left(g_{a}+i^{\mu }g_{b}+j^{\mu }g_{c}+k^{\mu }g_{d}\right)-\frac{1}{4}\frac{\partial f}{\partial q_{b}}\left(g_{a}+i^{\mu }g_{b}+j^{\mu }g_{c}+k^{\mu }g_{d}\right)i^{\mu }\\ & \;-\frac{1}{4}\frac{\partial f}{\partial q_{c}}\left(g_{a}+i^{\mu }g_{b}+j^{\mu }g_{c}+k^{\mu }g_{d}\right)j^{\mu }-\frac{1}{4}\frac{\partial f}{\partial q_{d}}\left(g_{a}+i^{\mu }g_{b}+j^{\mu }g_{c}+k^{\mu }g_{d}\right)k^{\mu }\\ & =f\frac{\partial g}{\partial q^{\mu }}+\frac{1}{4}\left(\frac{\partial f}{\partial q_{a}}g-\frac{\partial f}{\partial q_{b}}gi^{\mu }-\frac{\partial f}{\partial q_{c}}gj^{\mu }-\frac{\partial f}{\partial q_{d}}gk^{\mu }\right)\\ & =f\frac{\partial g}{\partial q^{\mu }}+\frac{1}{4}\left(\frac{\partial f}{\partial q_{a}}-\frac{\partial f}{\partial q_{b}}gi^{\mu }g^{-1}-\frac{\partial f}{\partial q_{c}}gj^{\mu }g^{-1}-\frac{\partial f}{\partial q_{d}}gk^{\mu }g^{-1}\right)g\\ & =f\frac{\partial g}{\partial q^{\mu }}+\frac{1}{4}\left(\frac{\partial f}{\partial q_{a}}-\frac{\partial f}{\partial q_{b}}i^{g\mu }-\frac{\partial f}{\partial q_{c}}j^{g\mu }-\frac{\partial f}{\partial q_{d}}k^{g\mu }\right)g=f\frac{\partial g}{\partial q^{\mu }}+\frac{\partial f}{\partial q^{g\mu }}g, \end{array}$$

where (2.4) was used in the second to last equality above, and definition 4.1 was used in the last equality. Hence, the first part of the theorem follows, and the second part can be proved in an analogous manner. ▪

## Appendix C. The proof of the chain rule

To prove the chain rule, we shall use the following lemma.

Lemma C.1 *Let q*=*q*_*a*_+*iq*_*b*_+*jq*_*c*_+*kq*_*d*_, *where* $q_{a},q_{b},q_{c},q_{d}\in R$. *Then, the partial derivatives of the quaternion composite function f*(*g*(*q*)) *satisfy the following chain rule*:

C 1 $$\begin{array}{rl} \frac{\partial f(\;g(q))}{\partial \xi } & =\frac{\partial f}{\partial g^{\nu }}\frac{\partial g^{\nu }}{\partial \xi }+\frac{\partial f}{\partial g^{\nu i}}\frac{\partial g^{\nu i}}{\partial \xi }+\frac{\partial f}{\partial g^{\nu j}}\frac{\partial g^{\nu j}}{\partial \xi }+\frac{\partial f}{\partial g^{\nu k}}\frac{\partial g^{\nu k}}{\partial \xi }\\ \;\text{and}\;\;\frac{\partial f(\;g(q))}{\partial \xi } & =\frac{\partial f}{\partial g^{\nu *}}\frac{\partial g^{\nu *}}{\partial \xi }+\frac{\partial f}{\partial g^{\nu i*}}\frac{\partial g^{\nu i*}}{\partial \xi }+\frac{\partial f}{\partial g^{\nu j*}}\frac{\partial g^{\nu j*}}{\partial \xi }+\frac{\partial f}{\partial g^{\nu k*}}\frac{\partial g^{\nu k*}}{\partial \xi }, \end{array}\}$$

*where ξ*∈{*q*_*a*_,*q*_*b*_,*q*_*c*_,*q*_*d*_} *and* ν∈H,ν≠0.

Proof. Let *g*(*q*)=*g*_*a*_+*ig*_*b*_+*jg*_*c*_+*kg*_*d*_, where $g_{a},g_{b},g_{c},g_{d}\in R$. Then, the function *f*(*g*(*q*)) can be seen as a function of the four real-valued variables *g*_*a*_,*g*_*b*_,*g*_*c*_ and *g*_*d*_, and the partial derivative of *f*(*g*(*q*)) can be expressed as

C 2 $$\frac{\partial f}{\partial \xi }=\frac{\partial f}{\partial g_{a}}\frac{\partial g_{a}}{\partial \xi }+\frac{\partial f}{\partial g_{b}}\frac{\partial g_{b}}{\partial \xi }+\frac{\partial f}{\partial g_{c}}\frac{\partial g_{c}}{\partial \xi }+\frac{\partial f}{\partial g_{d}}\frac{\partial g_{d}}{\partial \xi }.$$

By definition 4.1, the partial derivatives ∂*f*/∂*g*_*a*_, ∂*f*/∂*g*_*b*_, ∂*f*/∂*g*_*c*_ and ∂*f*/∂*g*_*d*_ are given by

C 3 $$\begin{array}{rl} \frac{\partial f}{\partial g_{a}} & =\frac{\partial f}{\partial g^{\nu }}+\frac{\partial f}{\partial g^{\nu i}}+\frac{\partial f}{\partial g^{\nu j}}+\frac{\partial f}{\partial g^{\nu k}},\\ \frac{\partial f}{\partial g_{b}} & =\left(\frac{\partial f}{\partial g^{\nu }}+\frac{\partial f}{\partial g^{\nu i}}-\frac{\partial f}{\partial g^{\nu j}}-\frac{\partial f}{\partial g^{\nu k}}\right)i^{\nu },\\ \;\frac{\partial f}{\partial g_{c}} & =\left(\frac{\partial f}{\partial g^{\nu }}-\frac{\partial f}{\partial g^{\nu i}}+\frac{\partial f}{\partial g^{\nu j}}-\frac{\partial f}{\partial g^{\nu k}}\right)j^{\nu }\\ \;\text{and}\;\;\frac{\partial f}{\partial g_{d}} & =\left(\frac{\partial f}{\partial g^{\nu }}-\frac{\partial f}{\partial g^{\nu i}}-\frac{\partial f}{\partial g^{\nu j}}+\frac{\partial f}{\partial g^{\nu k}}\right)k^{\nu }. \end{array}\}$$

Upon applying the quaternion rotation transform (⋅)^*ν*^ to both sides of (2.7) and replacing *q* with *g*, the real-valued components *g*_*a*_,*g*_*b*_,*g*_*c*_ and *g*_*d*_ can be expressed as

C 4 $$\begin{array}{rl} g_{a} & =\frac{1}{4}(g^{\nu }+g^{\nu i}+g^{\nu j}+g^{\nu k}),\;g_{b}=-\frac{i^{\nu }}{4}(g^{\nu }+g^{\nu i}-g^{\nu j}-g^{\nu k}),\\ \;g_{c} & =-\frac{j^{\nu }}{4}(g^{\nu }-g^{\nu i}+g^{\nu j}-g^{\nu k}),\;g_{d}=-\frac{k^{\nu }}{4}(g^{\nu }-g^{\nu i}-g^{\nu j}+g^{\nu k}). \end{array}\}$$

Taking the partial derivatives of both sides of the above equations yields

C 5 $$\begin{array}{rl} \frac{\partial g_{a}}{\partial \xi } & =\frac{1}{4}\left(\frac{\partial g^{\nu }}{\partial \xi }+\frac{\partial g^{\nu i}}{\partial \xi }+\frac{\partial g^{\nu j}}{\partial \xi }+\frac{\partial g^{\nu k}}{\partial \xi }\right),\\ \frac{\partial g_{b}}{\partial \xi } & =-\frac{i^{\nu }}{4}\left(\frac{\partial g^{\nu }}{\partial \xi }+\frac{\partial g^{\nu i}}{\partial \xi }-\frac{\partial g^{\nu j}}{\partial \xi }-\frac{\partial g^{\nu k}}{\partial \xi }\right),\\ \;\frac{\partial g_{c}}{\partial \xi } & =-\frac{j^{\nu }}{4}\left(\frac{\partial g^{\nu }}{\partial \xi }-\frac{\partial g^{\nu i}}{\partial \xi }+\frac{\partial g^{\nu j}}{\partial \xi }-\frac{\partial g^{\nu k}}{\partial \xi }\right)\\ \;\text{and}\;\;\frac{\partial g_{d}}{\partial \xi } & =-\frac{k^{\nu }}{4}\left(\frac{\partial g^{\nu }}{\partial \xi }-\frac{\partial g^{\nu i}}{\partial \xi }-\frac{\partial g^{\nu j}}{\partial \xi }+\frac{\partial g^{\nu k}}{\partial \xi }\right). \end{array}\}$$

Upon substituting the results from (C 3) and (C 5) into (C 2), and using the distributive law and merging similar terms, we finally have

C 6 $$\frac{\partial f(g(q))}{\partial \xi }=\frac{\partial f}{\partial g^{\nu }}\frac{\partial g^{\nu }}{\partial \xi }+\frac{\partial f}{\partial g^{\nu i}}\frac{\partial g^{\nu i}}{\partial \xi }+\frac{\partial f}{\partial g^{\nu j}}\frac{\partial g^{\nu j}}{\partial \xi }+\frac{\partial f}{\partial g^{\nu k}}\frac{\partial g^{\nu k}}{\partial \xi }.$$

Hence, the first equality of the lemma follows, the second equality following in a similar way. ▪

The proof of theorem 4.16 Using definition 4.1, the left HR derivative of the product *fg* can be expressed as

C 7 $$\frac{\partial f(g(q))}{\partial q^{\mu }}=\frac{1}{4}\left(\frac{\partial f(g(q))}{\partial q_{a}}-\frac{\partial f(g(q))}{\partial q_{b}}i^{\mu }-\frac{\partial f(g(q))}{\partial q_{c}}j^{\mu }-\frac{\partial f(g(q))}{\partial q_{d}}k^{\mu }\right).$$

Upon substituting the first equality of lemma C.1 into (C 7), it then follows that

C 8 $$\begin{array}{rl} \frac{\partial f(g(q))}{\partial q^{\mu }} & =\frac{1}{4}\left(\frac{\partial f}{\partial g^{\nu }}\frac{\partial g^{\nu }}{\partial q_{a}}+\frac{\partial f}{\partial g^{\nu i}}\frac{\partial g^{\nu i}}{\partial q_{a}}+\frac{\partial f}{\partial g^{\nu j}}\frac{\partial g^{\nu j}}{\partial q_{a}}+\frac{\partial f}{\partial g^{\nu k}}\frac{\partial g^{\nu k}}{\partial q_{a}}\right)\\ & \;-\frac{1}{4}\left(\frac{\partial f}{\partial g^{\nu }}\frac{\partial g^{\nu }}{\partial q_{b}}+\frac{\partial f}{\partial g^{\nu i}}\frac{\partial g^{\nu i}}{\partial q_{b}}+\frac{\partial f}{\partial g^{\nu j}}\frac{\partial g^{\nu j}}{\partial q_{b}}+\frac{\partial f}{\partial g^{\nu k}}\frac{\partial g^{\nu k}}{\partial q_{b}}\right)i^{\mu }\\ & \;-\frac{1}{4}\left(\frac{\partial f}{\partial g^{\nu }}\frac{\partial g^{\nu }}{\partial q_{c}}+\frac{\partial f}{\partial g^{\nu i}}\frac{\partial g^{\nu i}}{\partial q_{c}}+\frac{\partial f}{\partial g^{\nu j}}\frac{\partial g^{\nu j}}{\partial q_{c}}+\frac{\partial f}{\partial g^{\nu k}}\frac{\partial g^{\nu k}}{\partial q_{c}}\right)j^{\mu }\\ & \;-\frac{1}{4}\left(\frac{\partial f}{\partial g^{\nu }}\frac{\partial g^{\nu }}{\partial q_{d}}+\frac{\partial f}{\partial g^{\nu i}}\frac{\partial g^{\nu i}}{\partial q_{d}}+\frac{\partial f}{\partial g^{\nu j}}\frac{\partial g^{\nu j}}{\partial q_{d}}+\frac{\partial f}{\partial g^{\nu k}}\frac{\partial g^{\nu k}}{\partial q_{d}}\right)k^{\mu }. \end{array}$$

Grouping the terms ∂*f*/∂*g*^*ν*^, ∂*f*/∂*g*^*νi*^, ∂*f*/∂*g*^*νj*^ and ∂*f*/∂*g*^*νk*^ in (C 8) together, we have

C 9 $$\begin{array}{rl} \frac{\partial f(g(q))}{\partial q^{\mu }} & =\frac{\partial f}{\partial g^{\nu }}\frac{1}{4}\left(\frac{\partial g^{\nu }}{\partial q_{a}}-\frac{\partial g^{\nu }}{\partial q_{b}}i^{\mu }-\frac{\partial g^{\nu }}{\partial q_{c}}j^{\mu }-\frac{\partial g^{\nu }}{\partial q_{d}}k^{\mu }\right)\\ & \;+\frac{\partial f}{\partial g^{\nu i}}\frac{1}{4}\left(\frac{\partial g^{\nu i}}{\partial q_{a}}-\frac{\partial g^{\nu i}}{\partial q_{b}}i^{\mu }-\frac{\partial g^{\nu i}}{\partial q_{c}}j^{\mu }-\frac{\partial g^{\nu i}}{\partial q_{d}}k^{\mu }\right)\\ & \;+\frac{\partial f}{\partial g^{\nu j}}\frac{1}{4}\left(\frac{\partial g^{\nu j}}{\partial q_{a}}-\frac{\partial g^{\nu j}}{\partial q_{b}}i^{\mu }-\frac{\partial g^{\nu j}}{\partial q_{c}}j^{\mu }-\frac{\partial g^{\nu j}}{\partial q_{d}}k^{\mu }\right)\\ & \;+\frac{\partial f}{\partial g^{\nu k}}\frac{1}{4}\left(\frac{\partial g^{\nu k}}{\partial q_{a}}-\frac{\partial g^{\nu k}}{\partial q_{b}}i^{\mu }-\frac{\partial g^{\nu k}}{\partial q_{c}}j^{\mu }-\frac{\partial g^{\nu k}}{\partial q_{d}}k^{\mu }\right)\\ & =\frac{\partial f}{\partial g^{\nu }}\frac{\partial g^{\nu }}{\partial q^{\mu }}+\frac{\partial f}{\partial g^{\nu i}}\frac{\partial g^{\nu i}}{\partial q^{\mu }}+\frac{\partial f}{\partial g^{\nu j}}\frac{\partial g^{\nu j}}{\partial q^{\mu }}+\frac{\partial f}{\partial g^{\nu k}}\frac{\partial g^{\nu k}}{\partial q^{\mu }}. \end{array}$$

Hence, the first equality of the theorem follows, the other equalities following in a similar fashion. ▪

## Appendix D. Fundamental results based on the generalized HR derivatives

For convenience, several of the most important results for the left GHR derivatives are summarized in table 1. The symbols *ν*, *ω* and λ denote quaternion constants, *q* is a quaternion-valued variable and *μ* any quaternion constant or expression.
